# Modelling mixed crop-livestock systems and climate impact assessment in sub-Saharan Africa

**DOI:** 10.1038/s41598-024-81986-8

**Published:** 2025-01-09

**Authors:** Amit Kumar Srivastava, Jaber Rahimi, Karam Alsafadi, Murilo Vianna, Andreas Enders, Wenzhi Zheng, Alparslan Demircan, Mame Diarra Bousso Dieng, Seyni Salack, Babacar Faye, Manmeet Singh, Krishnagopal Halder, Frank Ewert, Thomas Gaiser

**Affiliations:** 1 Institute of Crop Science and Resource Conservation, University of Bonn, Katzenburgweg 5, D-53115 Bonn, Germany; 2 Leibniz Centre for Agricultural Landscape Research (ZALF), Eberswalder Str. 84, 15374 Müncheberg, Germany; 3 Karlsruhe Institute of Technology (KIT), Institute of Meteorology and Climate research (IMK-IFU), Kreuzeckbahnstrasse 19, 82467 Garmisch-Partenkirchen, Germany; 4 Department of Agroecology, Pioneer Center Land-CRAFT, Aarhus University, Aarhus, Denmark; 5 Key Laboratory of Marine Environmental Science, College of the Environment and Ecology, Xiamen University, Xiamen, 361102 China; 6 School of Geographical Sciences, Nanjing University of Information Science and Technology, Nanjing, 210044 China; 7 Department of Irrigation and Drainage Engineering, The School of Water Resource and Hydropower, Wuhan University, South East Lake Road 8, 430072 Wuhan, P.R. China; 8 WASCAL Competence Center (CoC), Blvd Moammar El-Kadhafi, Ouaga2000 06BP9507, Ouagadougou, Burkina Faso; 9 Université du Sine Saloum El Hadj Ibrahima NIASS, 55, Kaolack, Senegal; 10 Indian Institute of Tropical Meteorology, Ministry of Earth Sciences, Pune, 411008 India; 11 Centre of Excellence in Disaster Mitigation and Management (CoEDMM), Indian Institute of Technology, Roorkee, 247667 India

**Keywords:** Climate-change impacts, Plant sciences

## Abstract

Climate change significantly challenges smallholder mixed crop-livestock (MCL) systems in sub-Saharan Africa (SSA), affecting food and feed production. This study enhances the SIMPLACE modeling framework by incorporating crop-vegetation-livestock models, which contribute to the development of sustainable agricultural practices in response to climate change. Applying such a framework in a domain in West Africa (786,500 km^2^) allowed us to estimate the changes in crop (Maize, Millet, and Sorghum) yield, grass biomass, livestock numbers, and greenhouse gas emission in response to future climate scenarios. We demonstrate that this framework accurately estimated the key components of the domain for the past (1981–2005) and enables us to project their future changes using dynamically downscaled Global Circulation Model (GCM) projections (2020–2050). The results demonstrate that in the future, the northern part of the study area will likely experience a significant decline in crop biomass (up to -56%) and grass biomass (up to -57%) production leading to a decrease in livestock numbers (up to -43%). Consequently, this will impact total emissions (up to -47% CH_4_) and decrease of -41% in milk production, and − 47% in meat production concentrated in the Sahelian zone. Whereas, in pockets of the Sudanian zone, an increase in livestock population and CH_4_ emission of about + 24% has been estimated, indicating that variability in climate change impact is amplifying with no consistent pattern evident across the study domain.

**Supplementary Information:**

The online version contains supplementary material available at 10.1038/s41598-024-81986-8.

## Introduction

Smallholder mixed crop-livestock (MCL) farming systems hold significant importance in sub-Saharan African agriculture due to their widespread presence, livelihood provision, and ecosystem services^12,52^. These systems are under considerable pressure due to various challenges^6^. The livestock sector grapples with changes in quantity, quality, and distribution of feed resources, alterations in water resource availability as well as its quality, and diseases’ frequency^10,24,53^. Additionally, temperature variations caused by climate change contribute to heat stress among livestock^39^. The crop sector also faces unpredictable production outcomes as a result of frequent extreme events. Simultaneously, the interplay of interconnected factors, such as population growth, escalating food demand, expanding local and urban markets, and shifting dietary preferences driven by rising incomes, also exert substantial pressure on MCL systems, necessitating their adaptation and transformation to meet the evolving needs of society^35^.

While MCL farms have the potential to enhance farming efficiency and sustainability^51^, they often entail various trade-offs^17,28^. For example, using crop residues as fodder can help reduce feed gaps, but leaving the residues in the field allows for nutrient replenishment and improved soil fertility^8,36,54^. Therefore, to assess the performance of prevailing MCL practices and identify site-specific sustainable intensification strategies, it is essential to consider its multi-functionality and complex interactions^43^. Conducting observational assessments of different management options requires numerous field experiments, which are often limited by time and resource constraints. In contrast, process-based models offer a systematic approach to exploring various management scenarios on different spatiotemporal scales and can help identify suitable management strategies once they have been satisfactorily evaluated^32^. Crop models (CMs) and Livestock Models (LMs) can be used to simulate the combined effects of diverse management strategies on biomass, yield, water use, nutrient uptake (e.g., ^41,59^), and animal productivity, considering genotype, environmental interactions, and various herd characteristics (e.g., age, gender, and status; see^55^]). As an example of studies utilizing modeling tools to investigate the impact of climate change on forage availability, livestock, and crop productivity^13^ have employed the crop model APSIM^29^ and the livestock model Livsim^42^ within the AgMIP framework^40^.

However, in the majority of previous studies, CMs, LMs, and dynamic vegetation models (DVMs) are used as separate entities to investigate cropland, livestock, and rangeland conditions. While this approach enhances understanding of individual system components, it fails to capture the interactions at the landscape scale. To address this limitation, a coupled framework that combines all the components is necessary. The objective of this study is to employ the SIMPLACE modeling framework, which has been extensively tested and validated in the context of Sub-Saharan Africa^18,46^. In the present work, the SIMPLACE modelling framework was further extended with the new modules for simulating the mixed-crop-grass-livestock systems across Sahelian and Sudan savanna zones in Africa to assess the impacts of climate change on MCL farming systems. To this end, DynMod^34^, a livestock population model, has been combined with crop growth and grassland routines into an integrated model (IM) within the SIMPLACE framework. To better represent the Sahelian and Sudan savanna zones, two distinct model solutions were implemented within the SIMPLACE framework. The major difference in terms of grass simulations was the attribution of perennial grass types to the Sudan savanna and annual grass types to the Sahelian zone. In the Sudan savanna, distinct wet and dry seasons are experienced, and perennial grasses can survive the dry season and quickly regrow with the onset of rains, providing continuous ground cover and forage. Additionally, perennial grasses typically have deep root systems that allow them to access water from deeper soil layers, making them more drought-tolerant than annual grasses. Conversely, annual grasses were attributed to the Sahelian zone because they can thrive better within the short rainy season typical of this region. We chose millet and sorghum in one model implementation to represent the Sahelian zone because millet has a low water requirement compared to many other crops, making it highly drought-tolerant. This is crucial in the Sahelian zone, where rainfall is sparse and erratic. Conversely, a maize and sorghum combination was used to represent the Sudan savanna zone.

## Materials and methods

### Study area

**Fig. 1:**
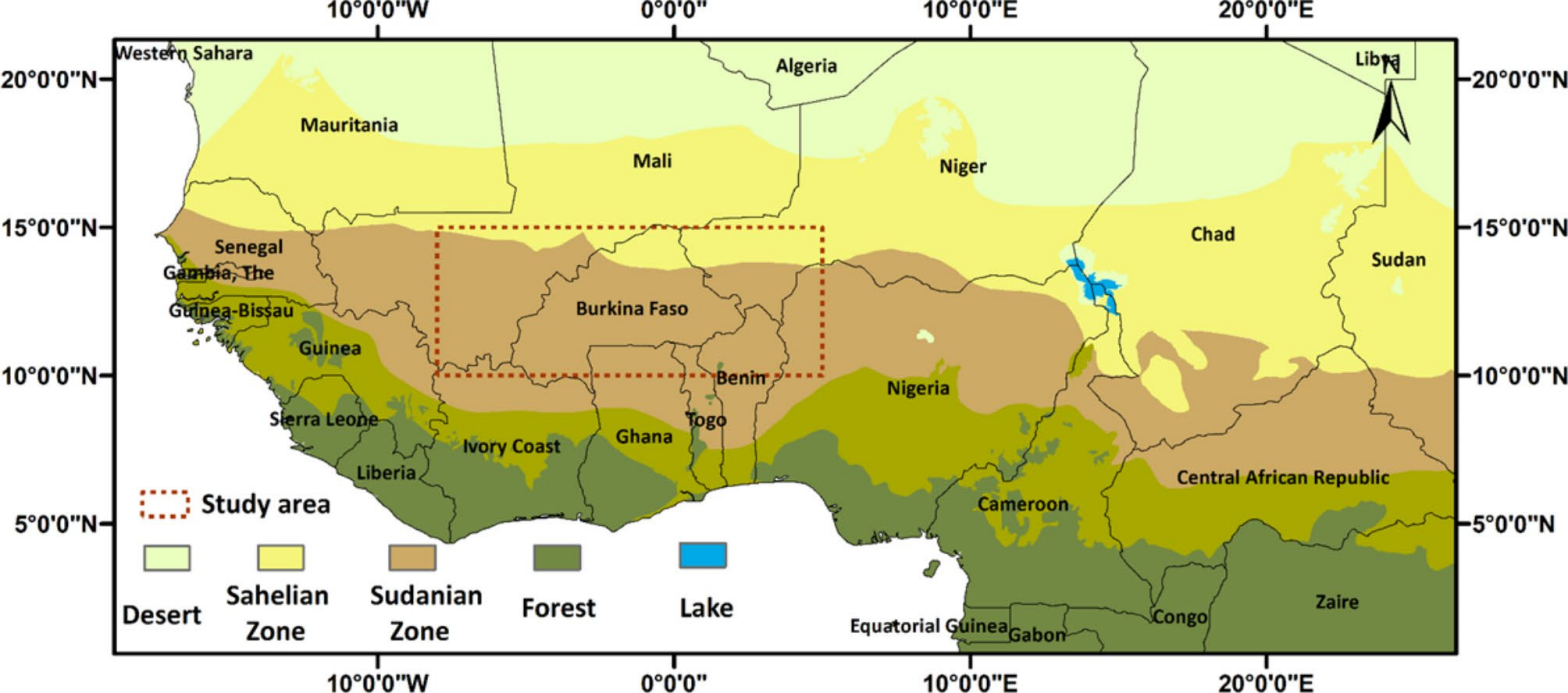
The geographical location of the studied domain and the agro-ecological zones of West Africa. (Map was created using qgis-3.22; https://download.qgis.org/).

The West African semiarid drylands span 11 countries, located between 15°E-16°W and 7–19°N, transitioning from forest to savanna and finally semi-desert grassland^31^. The region is divided into the Sahelian and Sudanian zones, differing in precipitation and dry season length. The Sahelian zone covers 1.3 × 10^6 km^2^, with temperatures ranging from 25 to 31 °C and annual precipitation between 150 and 600 mm. The Sudanian zone covers 1.7 × 10^6 km², with cooler temperatures (22–29 °C), higher precipitation (600–1200 mm yr^− 1^), and a shorter dry season. The majority of people in the area rely on agriculture, particularly extensive low-input pastoral and MCL farming systems that utilize natural pastures, crop residues, and browsing of trees and shrubs^2^. The geographical location of the studied domain (between a latitude of 10° and 15° N and a longitude of 08°E and 10°W) and the agro-ecological map of West Africa are shown in Fig. 1. The studied domain consists of 6500 grid cells of 0.1° × 0.1° size.

### The integrated modeling framework

#### Crops and grass growth simulation modules

**Fig. 2:**
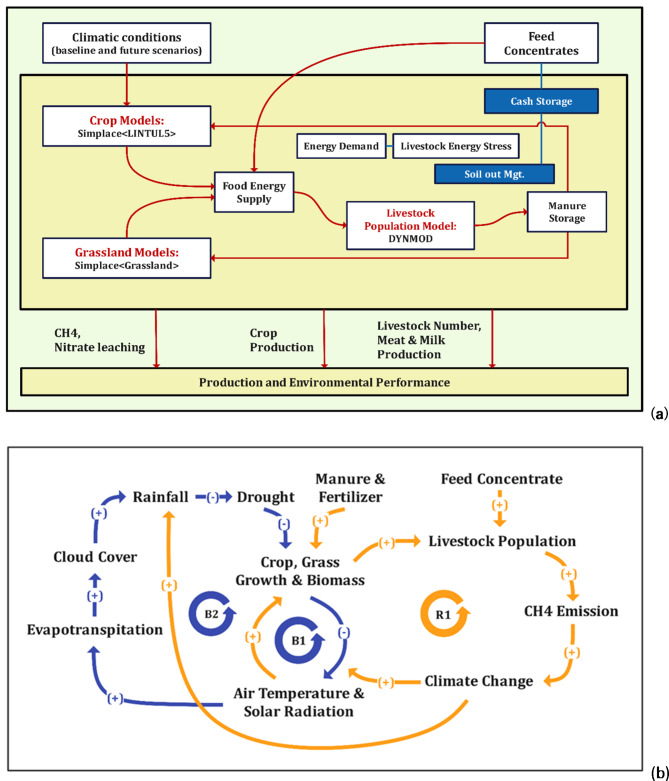
(**a**) Schematic presentation of the proposed Integrated model (IM) within the SIMPLACE Integrated modelling framework used in the current study. (**b**) A causal loop diagram (CLD) illustrating interactions and feedback loops in Crop-Livestock-Systems. R1 is the reinforcing loop, and B1, and B2 are the balancing loop. The plus (+) and Negative (-) sign indicates a positive and negative relationship between variables respectively. (Note: The dashed line connected from CH4 emission to Climate change shows a positive relationship (+), however, this has not been simulated in the current modelling framework).

In this study, we used the SIMPLACE < LINTUL5 > and SIMPLACE < Grassland > solutions combined into an integrated model within the SIMPLACE (Scientific Impact Assessment and Modelling Platform for Advanced Crop and Ecosystem Management) modeling framework to simulate crop yield and above-ground crop and grass biomass^16,20^ (Fig. 2). The LINTUL5 model within SIMPLACE estimates potential^57^, water-limited and nutrient-limited yields as well as above-ground biomass at a daily time scale. Potential yields are simulated under optimal conditions, considering solar radiation and temperature. Simulation of water-limited yield takes into account, in addition, limitations by temperature, radiation, water stress, and soil hydraulic properties, while nutrient-limited yield simulation incorporates additional nutrient limitations. Biomass production is determined by intercepted radiation and allocated to different crop organs based on developmental stage. Phenology is simulated by accumulating thermal time, and photosynthesis and crop growth rate are calculated using intercepted light and radiation use efficiency. The duration of the crop growth cycle is determined from emergence to physiological maturity. SLIM is a soil water balance model that estimates the daily change in soil water content in a variable number of soil layers based on the volumes of crop water uptake, soil evaporation, surface run-off, and seepage below the root zone. It allows for a more detailed representation of the soil profile by incorporating multiple soil layers, as opposed to simpler two-layer models. This enables better simulation of water movement, nutrient dynamics, and distribution throughout the soil column (https://simplace.net/doc/simplace_modules/net/simplace/sim/components/soil/slim/SlimWater.html), replacing the two-layer approach used in LINTUL5.

#### Livestock population growth simulation module

The module for simulating the growth of the livestock population calculates the daily changes in the number of animals across different age and gender groups. These groups include JuvenilesMale, SubAdultsMale, AdultsMale, JuvenilesFemale, SubAdultsFemale, and AdultsFemale. The module takes into account various factors such as birth rates, mortality rates, transition rates, intake rates, and off-take rates. The animals are categorized into six classes based on their age and gender, and the simulation focuses on the count of animals in each class and their transition processes, without considering individual animal age or weight. The birth rates are influenced by the “Parturition” input parameter, which can be adjusted using the “Prolification” fraction. The transition from one age class to another is determined by the “gamma” factor, which considers survival probabilities and the duration an animal remains in a particular class. The module also considers feed energy deficiency and mortality rates. The simulation for weight, meat, and milk production is based on the DYNMOD model^34^. It calculates the daily changes in live weight for each age and gender class, using the average weight of animals in each class. The model multiplies the number of animals in each class by their average live weight to calculate the live weight within a given class. The sum of the live weight of all classes sums up to the total live weight of the population. The change in live weight from one day to the next is determined by comparing the weights of animals on the current day with their weights on the previous day. Meat production is calculated by multiplying the live weight of the population by the carcass fraction. However, the actual selling of meat is not done within this module; it is handled in the livestock population growth simulation module. The number of animals sold can be a decimal fraction, as the module operates on a daily time step (more details in S6). The simulated crop residue and grass biomass are made available as a source of daily feed energy through the “Feed Energy Supply” module. This module calculates Livestock Energy Stress by comparing the daily feed energy demand to the feed energy supply, which ranges from 0 to 1 (0 = high stress, 1 = no stress). In case the feed energy supply from grass and crop residue biomass is insufficient to meet the livestock’s energy requirement, external feed concentrates can be provided. Farmers can decide, using the “Sell out Management” and “Cash Storage” components, whether to sell or keep the current number of livestock on a given day, depending on the availability of feed and their purchasing power for feed concentrates from the market (refer to S8). The manure produced by the livestock can be utilized as organic fertilizer either in grassland or cropland, depending on the farming-system management, which can be defined by the user. In the current IM, the estimation of crop yield, above-ground crop biomass, grass biomass, livestock population, meat and milk production, methane (CH4) emissions, and nitrogen loss (nitrate leaching) is possible (see Fig. 2a). Causal Loop Diagram (CLD) emphasizes the interconnected feedback loops and interactions between crops, grass, livestock, and climate change. The reinforcing and balancing loops are integral components that reflect the dynamic relationships within agricultural systems (Fig. 2b). It would help in identifying the critical points for intervention to improve system sustainability and productivity.

### Dataset used for model calibration at field scale

For the model calibration, the field experimental data comprising fertilizer application rates, phenological events, yield, and biomass of Maize, Millet, and Sorghum crops were obtained from the published data^18^. The grass biomass data at the field scale were obtained from Banizzoumnou and Sumbrugu in Niger and Ghana respectively^39^.

### Datasets used for model simulations at a regional scale

#### Climate data

In agricultural impact studies, it is necessary to apply bias correction to climate model outputs to accurately reproduce yields driven by observed climate^23^. In this study, a non-parametric trend-preserving quantile mapping (QM) approach, combined with multivariate bias correction (MBCn), was used to correct the climate data^7,14^. The approach considers the interdependency between climate variables during the historical period (1981–2005) and applies it to the near-future period (2023–2050). The study focuses on the near-future period and the RCP4.5 scenario, as it is less sensitive to uncertainties and benefits from high-resolution climate runs^15,27^. The West African Science Service Center on Climate Change and Adapted Land Use (WASCAL) project provides the high-resolution climate simulations used in this work^14,15,27^. The bias-correction method is applied to seven main climate variables for impact assessments: precipitation (Pr), mean near-surface air temperature (Tas), near-surface maximum air temperature (Tx), near-surface minimum air temperature (Tn), surface downwelling solar radiation (Rad), relative humidity (Rh, 2 m), and wind speed (Wd, 10 m). Climate change projections are generated by downscaling simulations from three global climate models (GCMs): MPI-ESM MR CCLM, HadGEM2-ES, and MPI-ESM WRF. The CCLM model follows the COordinated Regional-climate Downscaling EXperiment (CORDEX) domain setup^22,37,38^. The three models used in the current study herewith are as follows: M1 = CCLM-MPI-ESM; M2 = WRF-HADGEM2; M3 = WRF-MPI-ESM.

#### Soil data

The soil information necessary for the model includes pH, bulk density, organic carbon and nitrogen content (in kg kg^− 1^), soil texture (clay, silt, and sand content), and soil hydrological parameters (field capacity and wilting point in mm m^− 3^). This information was obtained from the ISRIC-WISE dataset (International Soil Reference and Information Centre-World Inventory of Soil Emission Potentials)^5^ for the center point of each grid cell.

#### Livestock population data

Livestock population dataset. The Gridded Livestock of the World version 3 (GLW3) database (available at http://www.fao.org/livestock-systems/global-distributions/en/) provides the livestock number (cattle, sheep, and goats) in each grid cell (with a spatial resolution of 5 arc-minutes). In the next step, for the period of the study (i.e. 1981–2005), this dataset was adjusted such that the absolute livestock population matches the official FAO’s sub-national estimates. It should be also noted that, since there was no measure for the temporal development of spatial livestock distribution, we assumed that the relative livestock distribution across the historical period was the same as the GLW3 database indicates for the year 2010.

#### Land use/cover data

This study uses three detailed land use and land cover (LULC) layers for the years 1975, 2000, and 2013, provided through the collaboration of CILSS (Comité permanent Inter-Etats de Lutte contre la Sécheresse dans le Sahel), USGS (United States Geological Survey), and USAID (United States Agency for International Development)^50^. These layers were created through expert interpretation of Landsat imagery, supplemented with aerial photographs, ASTER (Advanced Spaceborne Thermal Emission and Reflection Radiometer) imagery, and field observations^10,11^. The accuracy of these maps has been validated through multiple methods, including comparisons to historical imagery, field verification, and expert reviews, with overall accuracy reported to be around 73%^56^. Furthermore, studies such as those by^33^ have demonstrated that these LULC maps accurately represent land use history in the Mono River Basin, Togo. This dataset provides a reliable foundation for our analysis, offering both temporal consistency and the thematic detail essential for our modeling efforts. To achieve this, land-use types were quantified by counting the number of pixels within the 0.1° × 0.1° grid cells. The datasets represent the spatial distribution of various land-use classes within each grid cell, which were aggregated for this study into the share of grass-, crop-, tree-dominated areas, settlement areas, and others. The datasets cover the years 1975, 2000, and 2013. To establish representative land-use characteristics for the study period of 1981–2005, we calculated the average information across these three-time points. For the future time period (2020–2050), we assumed the same land use distribution as our baseline period (1981–2005).

#### Harvested area

The SPAM (Spatial Production Allocation Model) harvest area dataset was used to estimate the planted area of the three studied crops (i.e., maize, millet, and sorghum) in each grid cell. SPAM products are also subject to uncertainty, which varies from region to region and even from crop to crop^60^. have comprehensively evaluated this uncertainty, categorizing it into five levels (1 representing the lowest uncertainty, 5 the highest). Our domain falls, on average, into category 2 (ranging from 1 in Benin to 3 in Ivory Coast and Mali). It should also be noted that, since there was no measure for the temporal development of shifts in crop type cultivation within the cropland area, we assumed that the relative share of each crop planted in cropland areas across the historical period was the same as indicated in the SPAM database for the year 2010.

#### Crop varieties and crop calendar

Crop cultivars and grass types specific to the Sahelian and Sudanian agroecological zones were selected based on expert knowledge, their widespread adoption by farmers, and the availability of datasets for model cultivar calibration. To determine the simulated sowing date for each year, sowing windows and sowing rules were utilized^47^. The sowing windows defined the period when sowing could take place, while the sowing rules determined the actual day of sowing. According to the sowing rules, sowing would occur when the cumulative precipitation in the simulation unit over five days reached or exceeded 20 mm after the start of the sowing window. In cases where this condition was not met within a sowing window, sowing would be scheduled for the last day of that particular window.

#### Fertilizer usage dataset

Crop-specific fertilizer rates for different crops were obtained from^18^ and ^48^. No fertilizer was applied to millet and sorghum, while maize received 15 kg N ha^− 1^.

### Statistical analysis

The following indices have been used to assess the accuracy between observed and simulated data:

- RMSE comprises both precision and accuracy and has the same units as the variable of interest. Very sensitive to outliers.

$$\:\mathbf{R}\mathbf{o}\mathbf{o}\mathbf{t}\:\mathbf{M}\mathbf{e}\mathbf{a}\mathbf{n}\:\mathbf{S}\mathbf{q}\mathbf{u}\mathbf{a}\mathbf{r}\mathbf{e}\mathbf{d}\:\mathbf{E}\mathbf{r}\mathbf{r}\mathbf{o}\mathbf{r}\:\left(\mathbf{R}\mathbf{M}\mathbf{S}\mathbf{E}\right)\:=\:\sqrt{\frac{1}{n}\:\:\sum\:{\left({P}_{i}-{O}_{i}\right)}^{2}}$$

- Mean Residual Error (MR): Represents the percentage difference between simulated and observed data.

These indices serve as measures to evaluate the accuracy of the simulated values compared to the statistical (observed) data.

**Mean Residual Error (MR)** = $\:\frac{{S}_{i}-{O}_{i}}{{O}_{i}}\cdot\:100$

- Weighted Root Mean Square Error (WRMSE): Measure used to evaluate the accuracy of a predictive model, considering different weights for each observation.

**Weighted Root Mean Square Error (WRMSE)** = $\:\frac{{\sum\:}_{i=1}^{n}{w}_{i}\cdot\:{\left({P}_{i}-{O}_{i}\right)}^{2}}{{\sum\:}_{i=1}^{n}{w}_{i}}$

### Standardized water stress index

In order to quantify the sensitivity of biomass and crop yields to wet/dry conditions under the current climate and projections of climate change, the standardized water stress index (sWSI)^1^ was used. In this study, the sWSI was defined as the standardized difference between a plant’s loss of water (transpiration) and water supply (precipitation) as $\:\frac{WS-{WS}_{avg}}{{WS}_{Std}}$, where $\:WS$ is the difference between transpiration ($\:Ta$) and precipitation ($\:p$) ($\:WS=Ta-p$; in mm yr^−1^), during the crop and grass growing season, and $\:{WS}_{Std}$ and $\:{WS}_{avg}$indicate the standard deviation and multi-year average, respectively. This index demonstrates wet and dry conditions by tracking local water storage changes within the soil compared with water loss through transpiration.

### Gaussian process regression and causality analysis

The Gaussian process regression was carried out to test the non-linear dependency among all variables (i.e., temperature, precipitation, biomass, CH4, livestock, milk, and meat) for causality. The magnitude of each variable was analyzed using a Gaussian Process Regression (GPR) which is a type of Bayesian non-parametric regression. It is a probabilistic model that works well for both regression and classification. To estimate the noise level of data, a standard radial basis function kernel including a white kernel was applied. Furthermore, the hyperparameters of the kernel were optimized using log-marginal-likelihood (LML). To understand the complex data-driven causal relationships between all the variables, the Peters and Clark Momentary Conditional Independence (PCMCI) was performed. Peters and Clark Momentary Conditional Independence (PCMCI) is a causal discovery method for determining what factors in a dataset are causally related to one another. This technique has been developed for handling high dimensional data sets with time-dependent and non-linear relationships when aiming to estimate causal networks. It relies on the concept of temporary conditional independence, which holds that if two variables are causally coupled, then the inclusion of a third variable should not alter their mutual reliance. First, we identified a collection of potential explanatory factors to use the MCI method. Next, the algorithm employed a technique to check for temporary conditional independence between pairs of these variables. To do this, we estimated the mutual information between each pair of variables and then checked to see if it was substantially different from zero after adjusting for the other variables in the dataset. The existence of a causal link between two variables was shown by a non-zero value for the mutual information between them when all other variables were held constant. In this case, the direction of the mutual information indicates the direction of the causal link, with the variable that has greater mutual information being regarded as the cause and the variable that has lower mutual information being considered the consequence. A causal graph, where the edges reflect the associations between variables, can be constructed once all possible pairings of variables have been evaluated for momentary conditional independence. We used the “tigramite” library developed for Phyton Programming Language (Phyton Sofware Foundation. Phyton Language Reference, version 5.2 available at https://github.com/jakobrunge/tigramite).

## Results and discussion

After the development of the IM, we applied it to a specific domain in West Africa to investigate projected future climate changes. In this section, we discuss our findings regarding these climate projections and further explore the application of the IM to quantify the potential impacts on mixed crop-livestock systems.

### Projected future climate changes

**Fig. 3:**
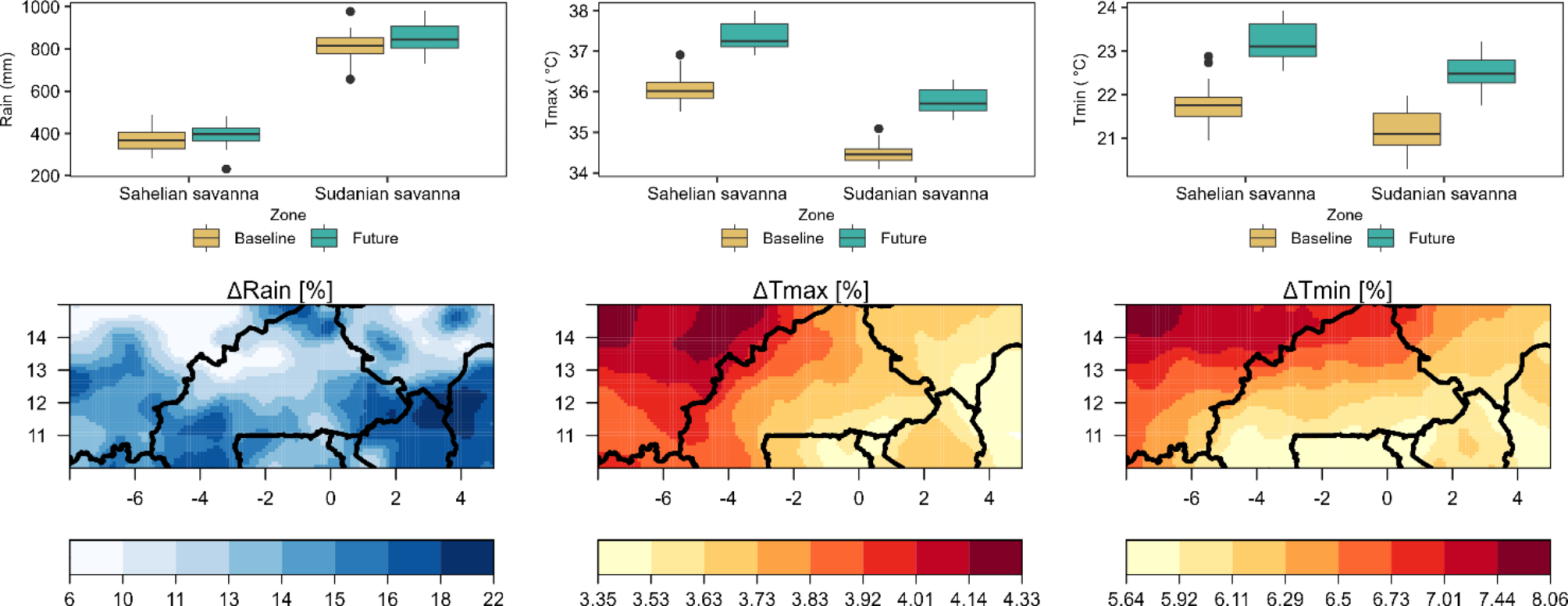
Mean and changes of rainfall, maximum temperature (Tmax), and minimum temperature (Tmin) are depicted in the top panels for baseline (1981–2005) and future (2023–2050) simulations in the Sahelian and Sudanian savanna regions. Colored ribbons indicate the standard deviation. The corresponding maps show the mean differences (%) between historical and future rainfall (ΔRain), Tmax (ΔTmax), and Tmin (ΔTmin) for the model ensemble (M1, M2, M3). Territorial borders are represented by solid black lines.

The upper section of Fig. 3 displays the averaged values from three GCM projections (M1, M2, and M3) for accumulated rainfall (Rain), highest temperature (Tmax), and lowest temperature (Tmin) in both the Sahelian and Sudanian savanna zones during the historical years (1981–2005) and the forthcoming timeframe (2023–2050). This figure also includes maps that visually represent the average percentage variations in annual rainfall, mean annual maximum, and minimum temperatures when comparing the historical and future periods.

#### Rainfall under historical and future scenarios

In the Sahelian zone, the historical coefficient of variation (CV) for accumulated annual rainfall over the grid cells fluctuated between 25.5% and 43.0%, while in the Sudanian zone, it ranged from 13.2 to 39.8%. The climate projection shows growth in this variability, with the Sahelian zone predicted to vary from 32.7 to 53.1%, and the Sudanian zone from 28.7 to 45.3%. This projection points to distinct differences in rainfall fluctuation between the past and projected future time frames. In the historical era from 1981 to 2005, the average accumulated rainfall was between 370.1 mm and 809.3 mm for the Sahelian and Sudanian zones, respectively. Looking to the future, from 2023 to 2050, the projections indicate a slight increase in these figures, with values ranging from 418.8 mm to 925.2 mm for both regions (Fig. 3). Moreover, compared to historical data, both the Sahelian and Sudanian zones are anticipated to see a rise in rainfall by 5.8–21.4% and 5.5–22.4%, respectively, across the area of study (Fig. 3). This pattern has previously been confirmed by Giannini et al.2019, Salack et al.,2022 in the Soudan/Sahel zone. For models M1, M2, and M3, the difference was between − 15% and + 35% (refer to Figure S2).

#### Maximum and minimum temperature under historical and future scenarios

Under both historical and future scenarios, the variations in maximum and minimum temperatures show distinct patterns. In the Sahelian zone, the coefficient of variation (CV) for the maximum temperature historically ranged from 1.6 to 2.4%, compared to 0.91–2.4% in the Sudanian zone. Future projections anticipate the CV in the Sudanian zone will be between 1.0% and 2.28%, while in the Sahelian zone, it’s expected to be between 1.6% and 2.3%. Concerning minimum temperature, the Sahelian zone experienced a historical spatial variability of 2.1–4.0%, whereas the Sudanian zone ranged from 1.9 to 4.8%. In the future, this is forecasted to vary from 2.1 to 3.3% in the Sahelian and 1.7–3.8% in the Sudanian zones.

The ensemble mean of different projections reveals for the period 2023 to 2050 an increase in the Sahelian zone’s maximum temperature between 1.26 °C and 1.47 °C (3.5–4.3%) and 1.14 °C to 1.55 °C (3.2–4.3%) in the Sudanian zone compared to historical records (Fig. 3). Similarly, an increase in minimum temperature is projected, ranging from 1.32 °C to 1.54 °C (6.0–7.4%) in the Sahelian zone and 1.19 °C to 1.63 °C (5.4–8.1%) in the Sudanian zone (Fig. 3). Our analysis reveals that temperatures will continue increasing, which exacerbates extremes heat such as hot days/nights^19^. On the other hand, it is well established that high temperatures will harm the growth, development, and yield of crops (Salack et al., 2016). For individual models M1, M2, and M3, the difference in CV was between 2.54 and 5.7% and + 8.45–5.16% in maximum and minimum temperature respectively (refer to Figure S2).

### Model calibration and evaluation

The model’s predictions at field scale for the yields of Millet (var. CIVT), Maize (var. EVDT97), and Sorghum (var. Kadaga) were found to be slightly overestimated by 2.1%, 1.0%, and 7.5%, respectively, in contrast to observed yields. The overestimation also extended to aboveground biomass, with excess predictions of 1.1%, 4.8%, and 2.0% respectively. While the harvest index (HI) was accurately estimated for the crops overall, the model slightly overestimated the HI of Millet and Sorghum by 1.0% and 5.4% respectively but underestimated the HI of Maize by -3.6% (Table S1).

With regard to grass biomass, the model predicted an overestimation in grass biomass dry matter (DM) by 16.4%, an RMSE and WRMSE value of 360.0 Kg ha^− 1^, and 342.1 Kg ha^− 1^ respectively, and an R^2^ value of 0.80.

**Fig. 4:**
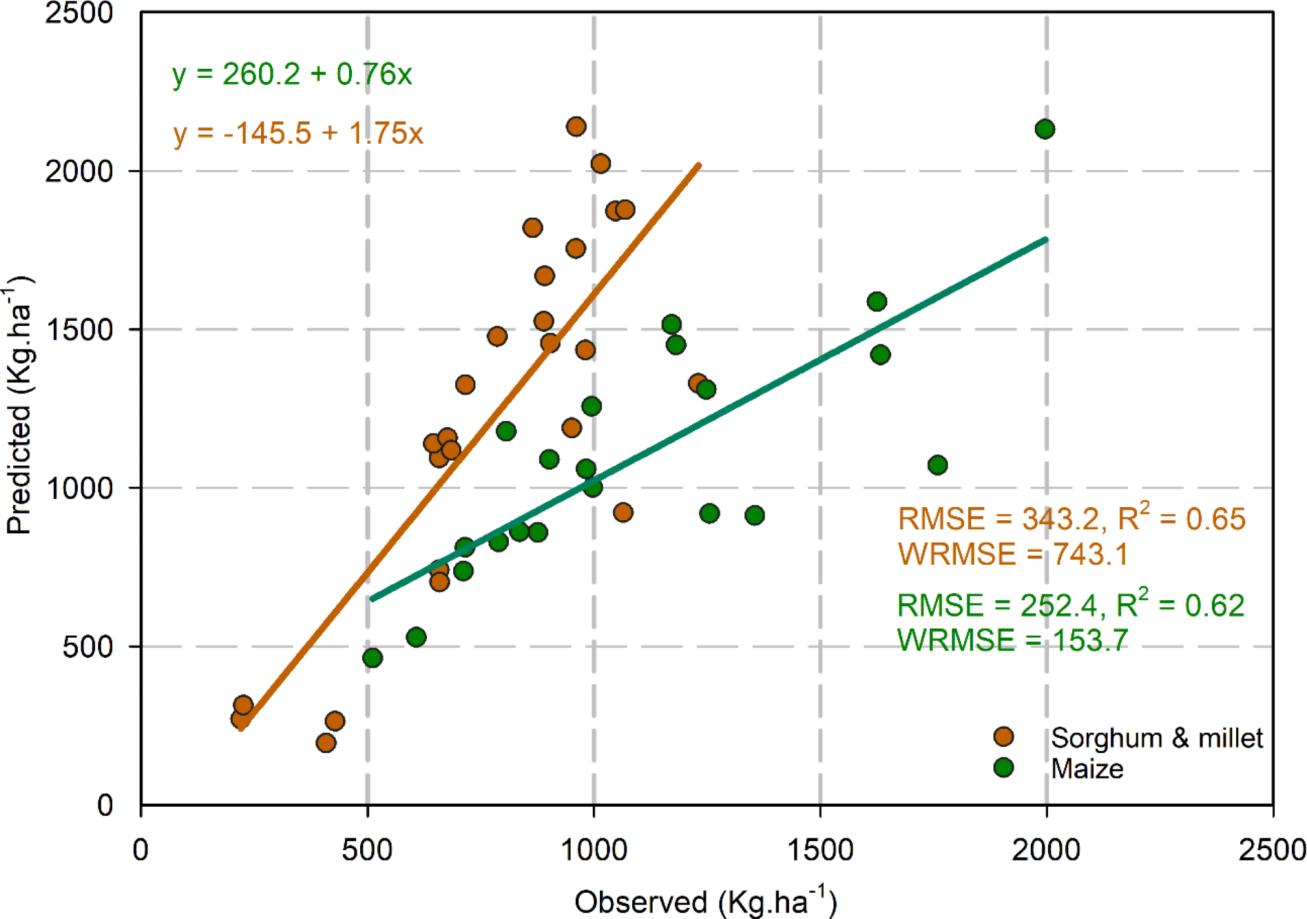
Comparison of observed and predicted Sorghum and Millet (for Sorghum, in 21 states, mean over 21 years from 1984–2004, and for Millet, in 4 states, mean over 20 years, from 1984–2002), Maize (in 21 states, mean over 4 years, from 2001–2004), yield dry matter (DM) in kg ha^− 1^ at states level in Burkina Faso.

**Fig. 5:**
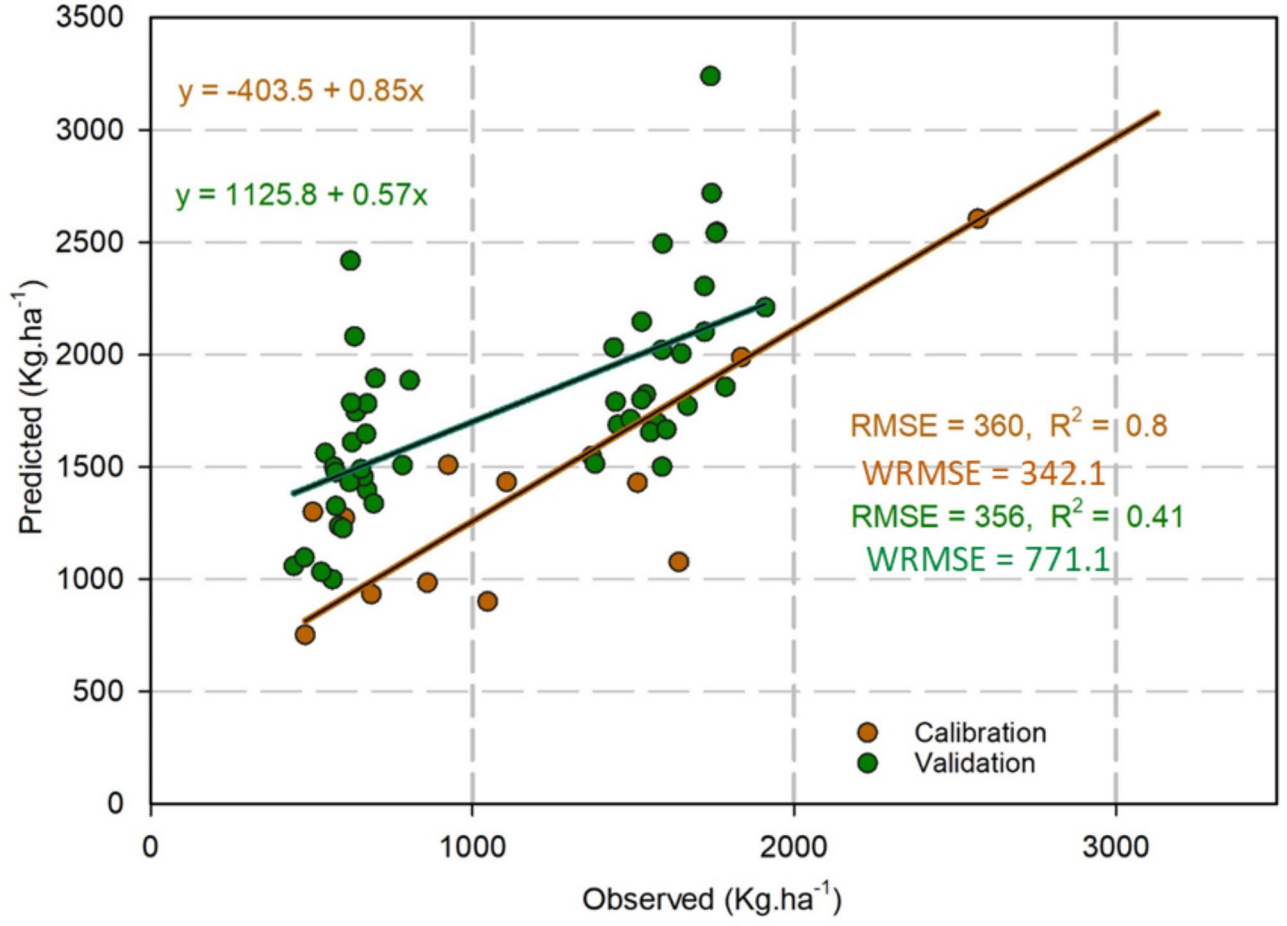
Comparison of observed and predicted grass biomass dry matter (DM) in kg ha^− 1^, at field scale for 13 years (‘Calibration’), and comparison of observed and predicted grass biomass dry matter (DM) in kg ha^− 1^ for 25 years (1981–2005) at regional scale covering the study transect (Validation).

At the transect scale in Burkina Faso, the model presented good accuracy for Maize, and Millet- Sorghum collectively across 21 states, with RMSE values of 252.4 Kg ha^− 1^, 343.2 Kg ha^− 1^ and WRMSE values of 153.7 Kg ha^− 1^ and 743.1 Kg ha^− 1^ respectively (Fig. 4), and an R^2^ value of 0.62 and 0.65 respectively. Conversely, the simulations for grass biomass dry matter at the transect level were overestimated with an RMSE and WRMSE of 356.0 Kg ha^− 1^ and 771.1 Kg ha^− 1^ respectively (Fig. 5), and an R^2^ value of 0.41.

**Fig. 6:**
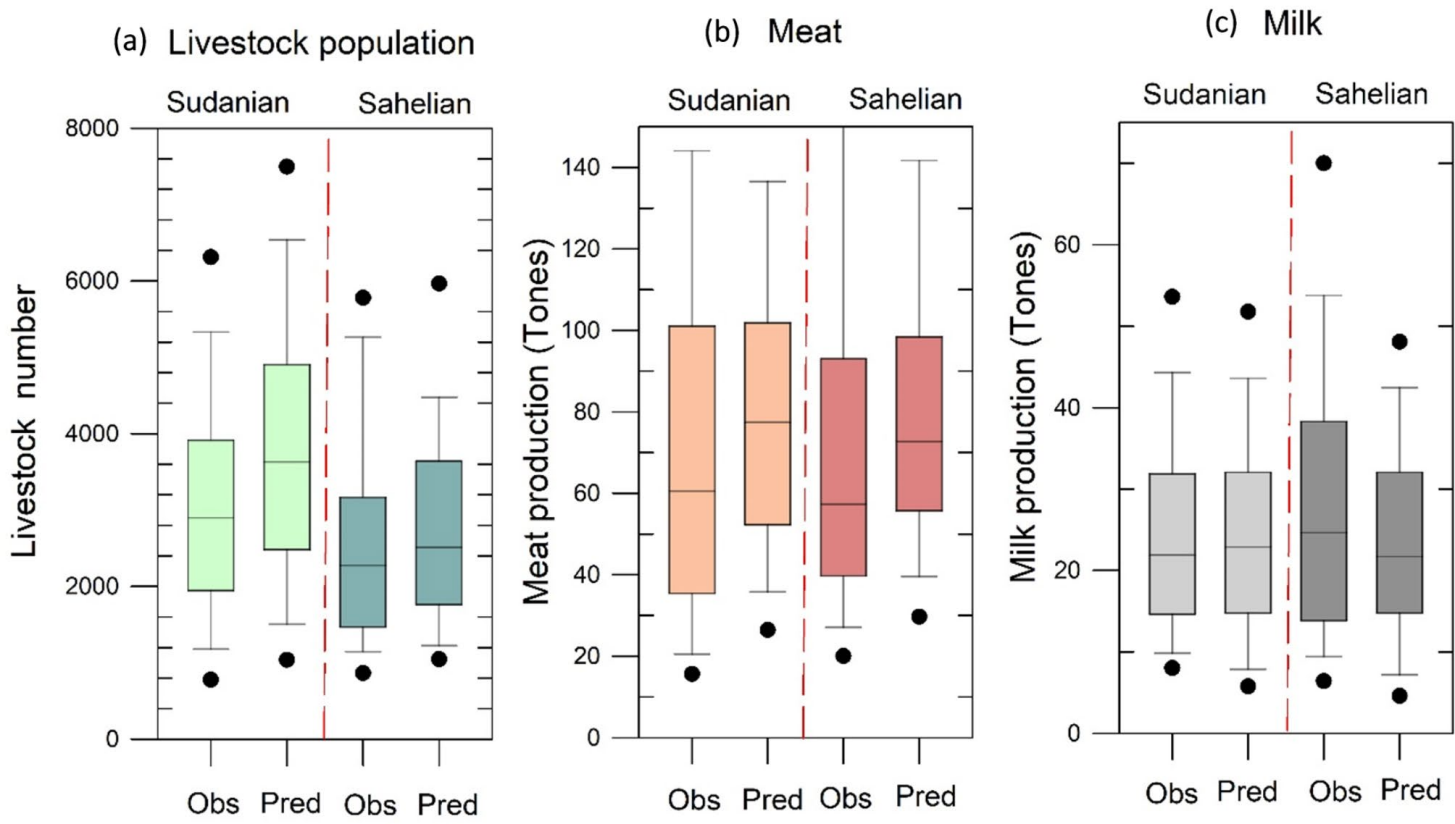
Comparison of observed and simulated livestock population in the year 2010 (**a**), Meat production (**b**), and Milk production (**c**) in the year 2000 at a regional scale covering the study transect.

For livestock population estimation, the model predicted numbers were 24.5% and 4.8% higher than the observed values in Sudanian and Sahelian zones respectively for the year 2010 (Fig. 6a). The milk production simulations were overestimated by 11.4% and 8.3% in the Sudanian and Sahelian zones respectively, compared to the observations for the year 2000 (Fig. 6b). Whereas simulations of meat production were underestimated by -2.4% and − 17.5% in Sudanian and Sahelian zones respectively, compared to the observations for the year 2000 (Fig. 6c).

### Crop yield and grass biomass under future climate scenarios

**Fig. 7:**
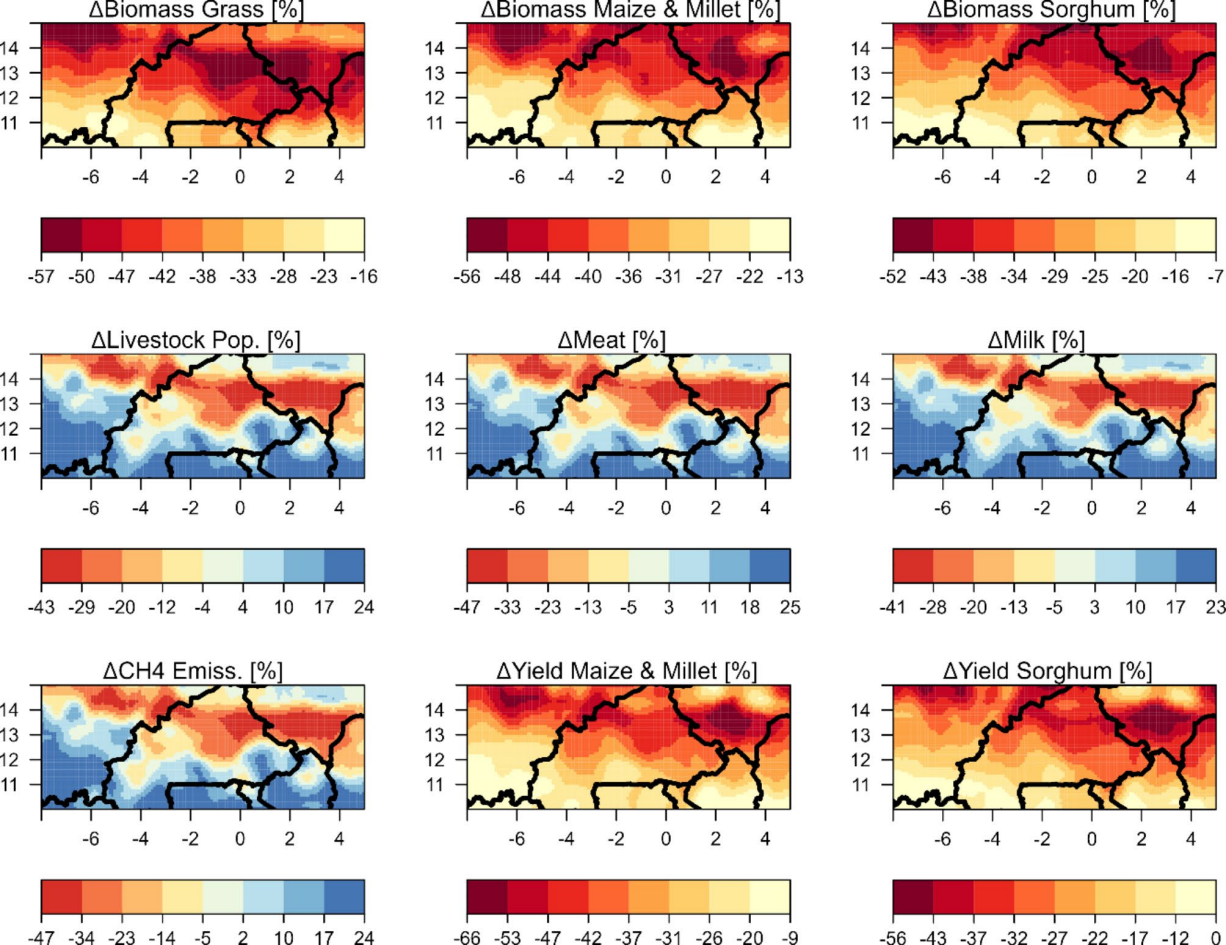
Mean differences (%) between historical (1981–2005) and future (2020–2050) simulations considering an ensemble of three GCMs for aboveground biomass produced by the grassland, maize and millet, and sorghum (top-row panels); livestock population, meat, and milk (center-row panels); and methane emissions (ΔCH4) from the integrated livestock system, and yield produced by maize and millet, sorghum (bottom-row panels). The solid black lines on the maps represent the territorial borders.

There was a significant change in the yield of maize in the Sudan savanna and millet in the Sahelian zone when comparing the historical and future time frames across the research transect. For models M1, M2, and M3, the yield difference was between − 88% and + 59% (Figure S5) depending on the crops and zones. However, when observed under the ensemble model, this difference lay between − 66% and − 9% (see Fig. 7).

The changes in sorghum yield for the predicted period ranged from − 75% to + 67% under models M1, M2, and M3. However, in the ensemble model, this range is adjusted to range between 0% and − 56%^18^. , however, predicted a decline of about 20% in future sorghum yields in the Sudan savanna. Their predicted loss for maize was around 15%, contrasting our prediction of a decrease up to -70.6%^45^. suggested an 11% decrease in millet yield by 2050 in the Sudan savanna, a less dramatic decrease than our − 78.2% estimate. A recent study by^48^, reported yield losses to the tune of 47% for short-cycle varieties and 44% for long-cycle varieties for RCP8.5, with the greatest impact in the Sahelian zone. Similar yield losses have been also found in previous studies using different crop models, such as SARRA-H model^49^, showed a decrease in millet and sorghum yields of about 0–41% in the 21st century over West Africa. A decrease in future maize yields was also found by^30^ and ^44^, reaching − 14% for sub-Saharan Africa by 2050, according to^30^, with impacts varying between − 30 and + 2% across sub-Saharan countries.

For maize and millet biomass, historical values in the Sahelian zone varied from 41.0 to 107.7% and from 14.6 to 220.7% in the Sudan savanna. Future projections place these values between 47.5 and 112.4% and 14.4–200.7%, respectively. The Sahelian zone showed maize and millet biomass differences of -10% to -74% using models M1, M2, and M3 (Figure S3) and − 13% to -56% in the ensemble model (Fig. 7).

Historical sorghum biomass varied from 39.3 to 93.0% in the Sahelian and 13.8–150.5% in the Sudan savanna. Predicted values range from 45.3 to 90.1% and 12.9–167.7% respectively. The Sahelian zone’s sorghum biomass difference under M1, M2, and M3 ranged from 0% to -68%, with the ensemble model varying between 52% and − 7% (Fig. 7).

Grass biomass historically ranged from 43.3 to 110.9% in the Sahelian and 18.3–102.7% in the Sudan savanna. Future values narrow down to 44.5–86.7% and 16.8–121.8% respectively. Using M1, M2, and M3, the Sahelian zone’s grass biomass difference spans − 67% to -10%, with the ensemble model differing from − 57% to -16% (Fig. 7). These outcomes highlight the biomass variability in the grass across periods and zones, which is further influenced by the model applied.

A trend of reduced crop durations was observed for Maize, Millet, and Sorghum, with grass significantly impacting yield and biomass reductions. In the Sahelian region, Millet’s growth period reduced by an average of five days across M1, M2, and M3. Future climates suggest Millet will experience greater water scarcity than in historical conditions. The Sudanian zone’s Maize growth period will reduce by about seven days. For Sorghum, the growth period will reduce by four days in the Sahelian and seven days in the Sudanian zone, with the Sahelian zone expecting harsher water scarcity.

Methane emissions in both the Sahelian and Sudan savanna zones exhibited variability. Historical values ranged between 31.5 and 41.9% and 24.0–48.4%, respectively. Future projections suggest values of 24.0–48.4% in the Sahelian and 15.6–89.5% in the Sudan savanna. The Sahelian zone, under M1, M2, and M3, showed a methane emission difference of + 39% to -60% (Figure S5), while the ensemble model differed by + 24% to -47% (Fig. 7).

### Sensitivity of yields and AGB to sWSI’s wet/dry conditions

To quantify the sensitivity of yields and aboveground biomass (AGB) availability for crop and grass to sWSI variability, the study utilized yearly model simulation outputs at a spatial resolution of 0.1 arc degrees for both current climate conditions (referred to as “His”) and projected climate change scenario (RCP4.5).

Regarding the sensitivity of maize and millet yields to sWSI variability under current climate conditions, the results showed that only 2.1%, 4.5%, and 0.9% of the study area exhibited significant positive correlation values for M1, M2, and M3 models, respectively (Figure S6). On the other hand, under the projected climate change scenario, 21%, 0.2%, and 10.4% of the study area showed significantly negative correlation values for the M1, M2, and M3 models, respectively. These percentage values confirmed that wet stress conditions were more dominant than dry stress conditions based on historical data, specifically in the northern part of the study area according to M1, and the southern part according to M3. However, the response mechanism to dry stress was reversed under the climate change scenario, where 3.6%, 12.8%, and 14.8% of the study area exhibited significantly positive correlation values for M1, M2, and M3 models, respectively, with remarkable increases. The same patterns were predicted for the sensitivity of sorghum yields to sWSI variability under current climate conditions but, interestingly, for the M3 model, 28.1% of the study area exhibited a significantly negative correlation which confirmed that wet stress conditions were more dominant than dry stress conditions (southern region) but this response becomes opposite under climate change where 28.1% of study area exhibited significantly positive correlation which confirmed that dry stress conditions were more dominant in the future.

**Fig. 8:**
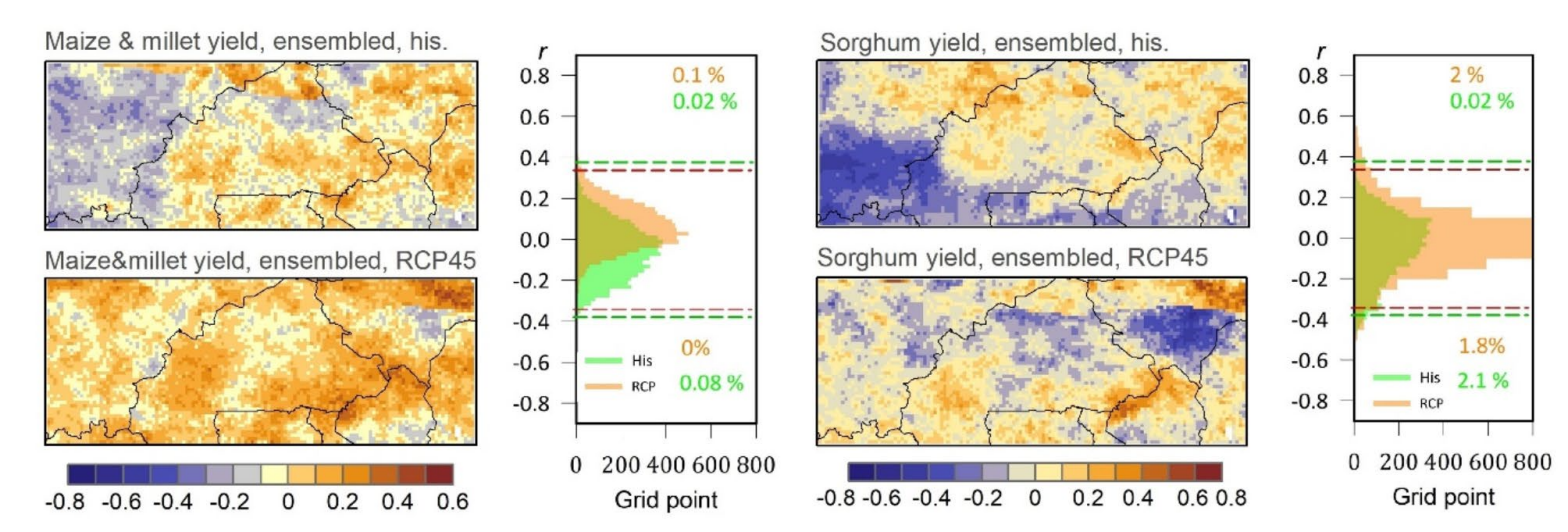
Spatial correlation between the crop yields and the annual sWSI for both ensembled historical climate conditions (“His”) and the ensembled future conditions (RCP4.5). In the histograms of the frequency distribution of correlation coefficients for historic (red) and future (green) conditions, the red and green dashed lines represent the critical values of correlation at a significant level of *p* < 0.05 for the “His” and RCP4.5 datasets, respectively.

**Fig. 9:**
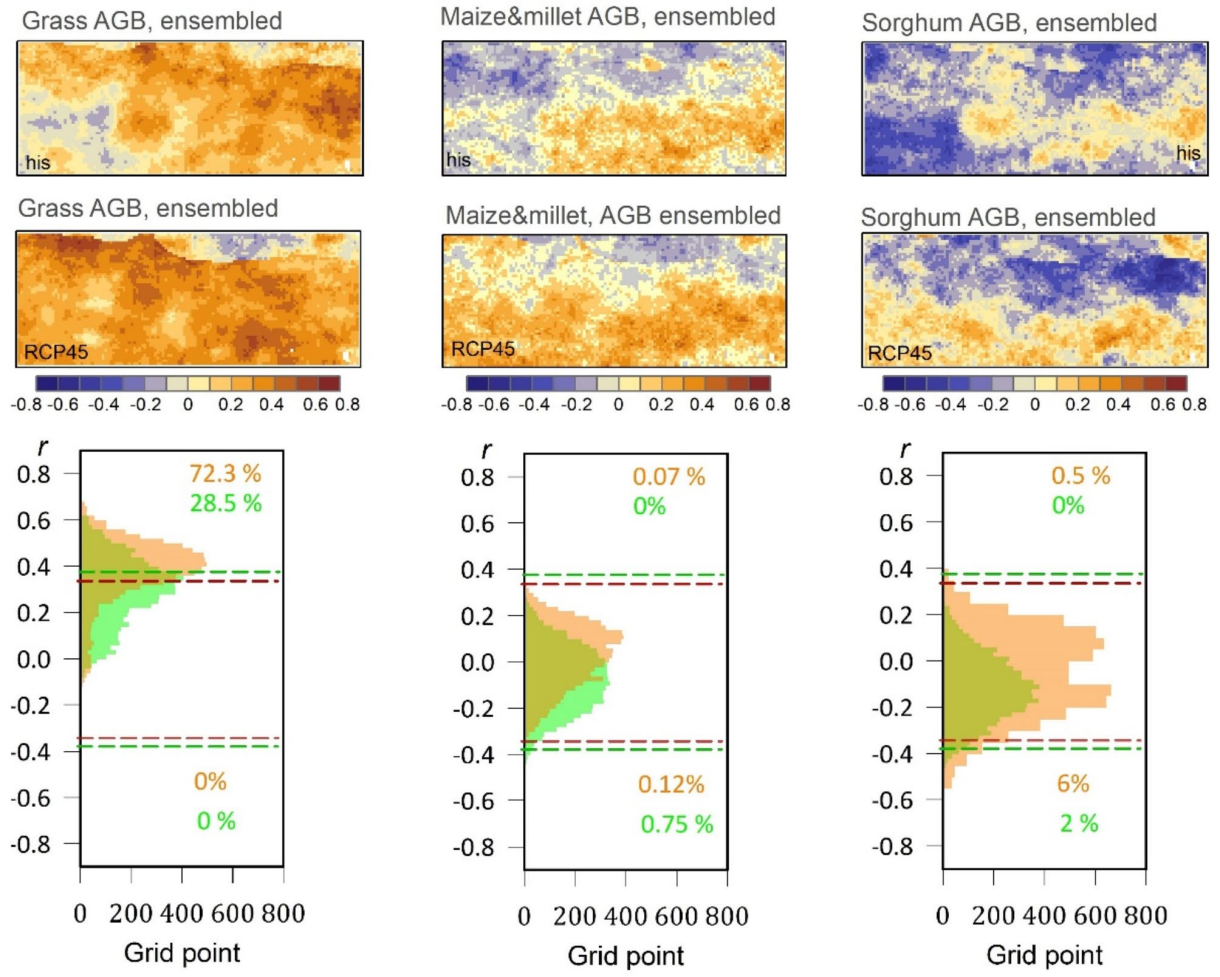
Spatial correlation between the AGB of grass and crops and the annual sWSI for both historical climate conditions (“His”) and the projected future scenario (RCP4.5). In the histograms of the frequency distribution of correlation coefficients for historic (red dashed lines) and future (green dashed lines) conditions, the red and green dashed lines represent the critical values of correlation at a significant level of *p* < 0.05 for the “His” and RCP4.5 datasets, respectively.

As depicted in Figure S7, correlation analysis was conducted for grassland ecosystems, revealing a highly significant positive correlation between grass AGB and annual sWSI for West Africa. The results showed that under current climate conditions, 29.8%, 65.5%, and 34.9% of the study area exhibited significantly positive correlation values for the M1, M2, and M3 models, respectively. These values became more pronounced when considering projected climate change (RCP4.5), with 39.1%, 73.3%, and 80.8% of the study area showing significantly positive correlation values for M1, M2, and M3 models, respectively. This indicates an increase of + 9.3%, + 7.8%, and + 45.9% compared to the current climate. Figures 8 and 9 indicate a spatial correlation between the crop yields and AGB and the annual sWSI under the ensemble model for the current and projected climate. Where 28.5%, and 72.3% of the study area’s AGB showed significantly positive correlation values against the annual sWSI for current and projected climate, respectively. Similar relationships between wet conditions and crop yields have been estimated in other studies, including investigations of hot extremes in Burkina^9^.

The regions with the highest significant positive correlations under the current climate were predominantly located in the eastern, southern, and central parts of the study area, particularly in the east of Burkina Faso, the central area of Benin, and some parts of western Nigeria, for M1 model. For the M2 model, the highest significant positive correlations extended to southern Niger and most of Burkina Faso under both current and projected climate change scenarios. The results indicated that the AGB output of the M3 model demonstrated greater sensitivity to dry stress conditions compared to wet stress conditions. Although there were variations in the spatial patterns of AGB responses among the models, they all consistently highlighted the presence of intense dry stress conditions.

Regarding the sensitivity of maize and millet AGB to sWSI variability under current climate conditions (Figure S7), the results indicate that only 1%, 6.9%, and 0.1% of the study area demonstrate significant positive correlation values for M1, M2, and M3 models, respectively. On the other hand, 27.7%, 0%, and 9.8% of the study area exhibit significantly negative correlation values for the M1, M2, and M3 models, respectively. These percentage values confirm that wet stress conditions were more dominant than dry stress conditions based on historical data, particularly in the northern part of the study area according to the M1 model, and the southern and western parts according to the M3 model. However, the response mechanism to dry stress is reversed under the climate change scenario, where 1.8%, 10.5%, and 10.5% of the study area display significantly positive correlation values for M1, M2, and M3 models, respectively, indicating remarkable increases (Figure S7).

Interestingly, according to the M1 model, there is a significant negative correlation between sorghum AGB and the sWSI. Approximately 28.5% and 35.6% of the study area exhibited significant negative correlation values for both current climate conditions and the projected climate change scenario, RCP45, respectively. These percentages indicate that wet stress conditions are more dominant than dry stress conditions, and this dominance is expected to increase further in the future, particularly in the northern and western parts of the study area. The presence of dry stress conditions (indicated by positive correlation values) was relatively limited according to the M3 model and the RCP4.5 scenario, affecting only 7.3% of the study area (Figure S7).

### Livestock number, meat, and milk production under future climate scenarios

In the Sahelian region, livestock numbers varied, with past figures spanning 13.9–37.1%, and future estimates from 14.3 to 37.7%. In the Sudan savanna area, historical livestock numbers were between 20.5% and 182.2%, while future projections spanned 8.4–170.1%.

In the Sahelian region, the difference in livestock numbers between the two periods varied. Based on models M1, M2, and M3, variations ranged from − 55% to + 44% (Figure S4). However, in the ensemble model, this span was between − 43% and + 24% (Fig. 7).

In the realm of milk production, the Sahelian zone displayed fluctuations, with past data between 10.2% and 34.0%, and future predictions between 12.5% and 36.6%. The Sudan savanna zone also showed changes in milk production, historically varying between 17.0% and 154.9%, and in the future, from 6.7 to 156.2%.

For milk production in the Sahelian zone, models M1, M2, and M3 displayed differences from − 52% to + 38% (Figure S4). The ensemble model indicated a variation from + 23% to -41% (Fig. 7).

Regarding meat production, the Sahelian region’s historical figures varied between 34.3% and 45.2%, while future estimates ranged from 25.8 to 45.8%. In the Sudan savanna, historical meat production ranged from 28.2 to 65.6% and future projections spanned 14.7–84.4%. The difference under models M1, M2, and M3 was between + 41% to -60% (Figure S4). The ensemble model demonstrated a variation from − 47.0% to -25%, as highlighted in Fig. 7.

This paper explores uncertainties in the multi-model ensemble of General Circulation Models (GCMs) from the CMIP5 by considering diverse GCM datasets for each climate parameter. The findings presented herein are based on the RCP4.5 scenario, which envisions a trajectory where greenhouse gas emissions rise and then peak around the mid-21st century. Post-peak, emissions gradually decrease due to the implementation of mitigation policies and technologies^58^. Given our reliance on RCP4.5, it’s important to note that results may differ under alternative Representative Concentration Pathways (RCPs). Previous research indicates that RCP8.5, characterized by high emissions, could lead to a sustained vulnerability increase in food security, particularly in vulnerable regions like West Africa. Despite substantial adaptation investments, these impacts on food security associated with high emissions may be challenging to counteract. However, the plausibility of the RCP8.5 scenario is debated due to concerns about the availability of coal required to align with high greenhouse gas emissions^25,26^].

This paper also assesses livestock exposure to future cumulative stressors using the static GLW3 livestock spatial distribution for the year 2010. Notably, the livestock data lacks information on seasonal dynamics/displacements of animals or projections of future livestock evolution, which could influence our results. Previous studies have projected an anticipated decline of 7.5 to 9.6% in global livestock for the year 2010 due to diminishing herbaceous production, with the most significant impact expected in sub-Saharan Western Africa. Our findings align with this upward trend in livestock risk until the 2050s, indicating a potential decrease of -43% in the Sahelian zone due to declining grass biomass production. Conversely, in the Sudanian zone, the livestock population is expected to increase by up to 24%, primarily attributed to the augmented feed availability resulting from increased grass biomass production.

**Fig. 10:**
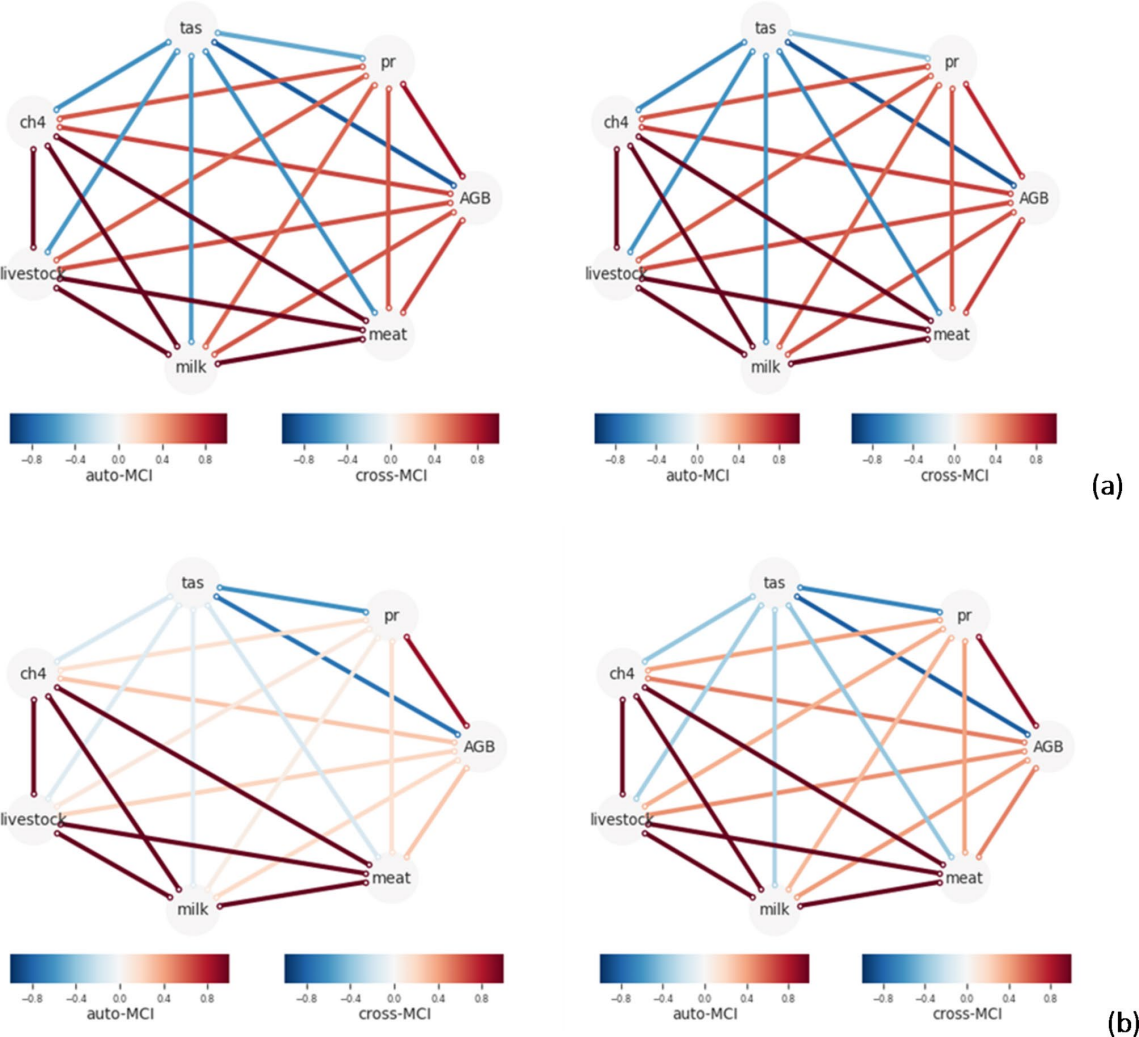
(**a**) Causal discovery graph generated for the Sahelian region using PCMCI showing the relationships of variables (i.e. mean temperature (tas), precipitation (pr), biomass (AGB), methane emission (ch4), livestock, milk, and meat). The node colors indicate the nonlinear auto-dependency of each variable (auto-MCI). The link colors indicate the interdependency strength (cross-MCI) between variables. (**b**) Causal discovery graph generated for Sudan savanna region using PCMCI showing the relationships of variables (i.e. mean temperature (tas), precipitation (pr), biomass (AGB), methane emission (ch4), livestock, milk, and meat). The node colours indicate the nonlinear auto-dependency of each variable (auto-MCI). The link colors indicate the interdependency strength (cross-MCI) between variables.

The causal analysis of the evolving climate landscape depicts the relationships between livestock, temperature, and precipitation showing substantial changes in Sahelian and Sudan savanna zones (Fig. 10a and b). Historically, livestock have exhibited a negative relationship with temperature (strength=-0.119, max value=-1/1), meaning that higher temperatures have been associated with a decrease in livestock numbers. This negative relationship is expected to intensify (strength= -0.335, max value= -1/1), suggesting that increased heat will further reduce livestock populations.

Consequently, this trend indicates a decline in the heat resilience of livestock in a future, warmer world. Moreover, the relationship strength between livestock, above-ground biomass (AGB) (from strength = 0.187 in the past to 0.447 in future, max value= -1/1), and rainfall (from strength = 0.103 in the past to 0.355 in future) is expected to more than double. This change suggests an increasing demand for feed and water resources, coupled with a decline in the heat resilience of livestock in the future. The enhanced relationship between AGB, rainfall, and livestock signifies a greater dependency on these resources, which could exacerbate vulnerabilities in the face of climate variability. However, it is important to note that the relationships between livestock and key productivity metrics such as meat, milk, and emissions are projected to remain consistent in the future. This stability implies that, despite the adverse impacts of temperature and precipitation, the production outputs and emissions associated with livestock may not experience significant changes.

**Fig. 11:**
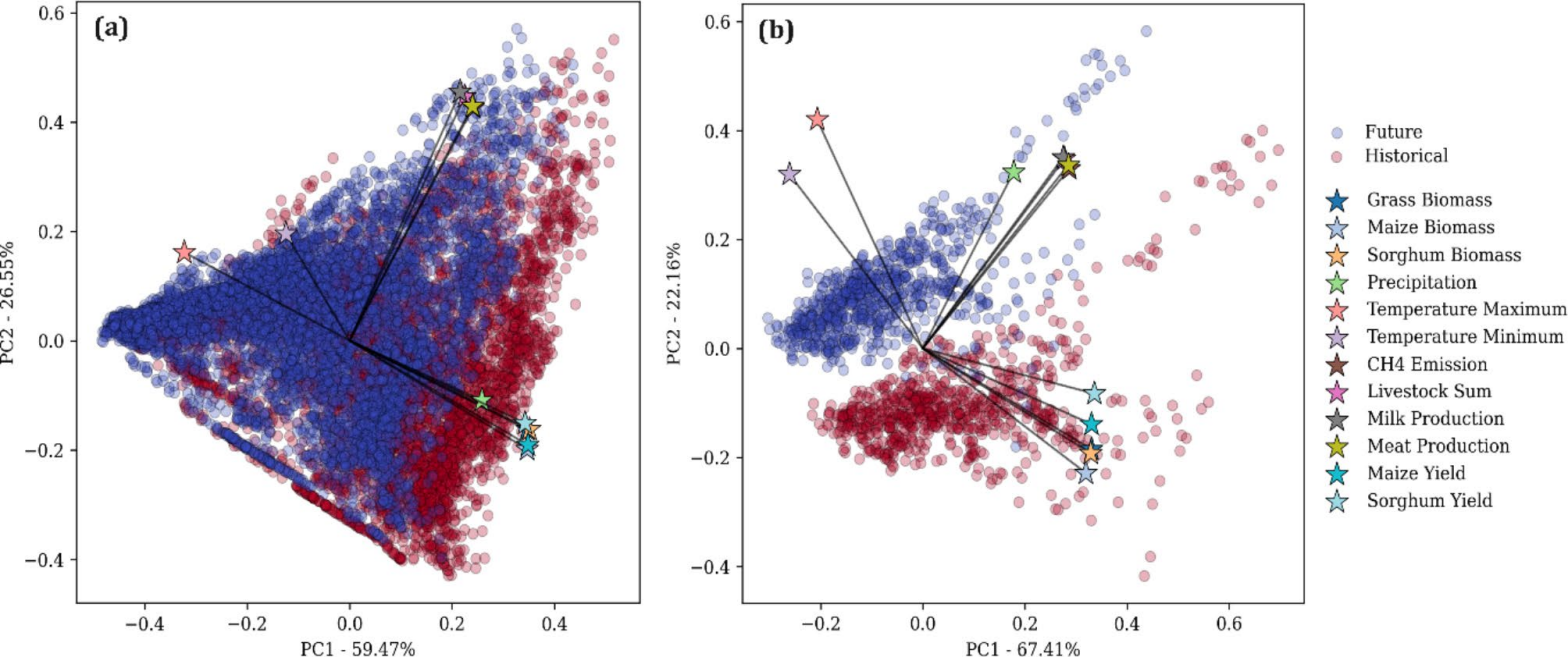
Illustration of Principal Component Analysis (PCA) applied to crop and grass yields, biomass, and various climatic variables (maximum and minimum temperatures, precipitation), as well as livestock numbers, meat and milk production, and methane (CH_4_) emissions, under historical and future scenarios in Sudan savanna (**a**), and Sahelian zone (**b**). The biplots depict the distribution of data points within the principal component space. Historical data points are represented in red, while future data points are shown in blue. Vectors indicate the direction and strength of the relationship between the principal components and the original variables.

The PC1 (59.47%) and PC2 (26.55%) together (Fig. 11) explain a significant portion of the total variance (86.02%) in the data from the Sudan savanna. In the Sahelian zone, PC1 (67.41%) and PC2 (22.19%) account for 89.6% of the total variance. Grass biomass, maize biomass, sorghum biomass, and precipitation have strong positive loadings on PC1, indicating their substantial contribution to the first principal component. Maximum temperature, minimum temperature, and CH4 emissions exhibit strong positive loadings on PC2, highlighting their influence on the second principal component. Livestock sum, milk production, maize production, maize yield, and sorghum yield also show significant loadings, demonstrating their relevance to both PC1 and PC2. Future scenarios (blue points) tend to cluster towards the positive end of PC1, indicating increased values for biomass production and precipitation. Historical scenarios (red points) are more dispersed along PC2, reflecting greater variability in temperature and CH4 emissions. The alignment of grass biomass, maize biomass, and sorghum biomass vectors with PC1 underscores the influence of biomass production on this principal component, suggesting that future changes in biomass production will be a major driver of variance. The vectors for maximum temperature, minimum temperature, and CH4 emissions aligned with PC2 indicate that these climatic factors significantly impact the second principal component, emphasizing their importance in future scenarios. The distinct clustering and separation of data points suggest potential shifts in agricultural production and climatic conditions under future scenarios, highlighting areas that may require adaptive strategies to mitigate adverse impacts.

## Conclusions and limitations

In conclusion, this research study provides insights into the model’s predictions and their implications across various agricultural and environmental parameters. The findings indicate that the model slightly overestimated the yields of Millet, Maize, and Sorghum by specific percentages. Although the overall accuracy of the harvest index (HI) estimations was satisfactory, the model displayed deviations for individual crops, with slight overestimations for Millet and Sorghum and an underestimation for Maize. Furthermore, the study underscores the model’s performance at the transect scale, demonstrating good accuracy in predicting crop yields for Maize, Millet, and Sorghum across different regions. However, predictions for grass biomass dry matter at this scale were overestimated.

In the realm of livestock estimation, the model’s predictions deviated from observed values, overestimating numbers while underestimating meat and milk simulations. This highlights the complexity of capturing livestock dynamics accurately. The research also delves into the ensemble model’s variability, showcasing the wide range of yield fluctuations in historical and projected periods, especially concerning maize, millet, and sorghum. Additionally, the study emphasizes the considerable influence of changing climate conditions on crop durations, with potential impacts on yields and biomass.

Methane emissions exhibited variability across regions, both historically and in projections. The model’s predictions indicated shifts in emissions influenced by changing conditions, with variations in estimates under different scenarios.

Despite these unique features, the study also suffers from limitations. Modeling framework SIMPLACE, used herein, to examine climate change effects on smallholder mixed crop-livestock (MCL) systems in sub-Saharan Africa (SSA), depends on multiple sources of gridded data, each presenting its uncertainties. Climate data, for instance, is subject to bias correction techniques such as non-parametric trend-preserving quantile mapping (QM) and multivariate bias correction (MBCn) to accurately simulate yields based on observed climate. These methods preserve the interdependencies between climate variables but rely on assumptions that can introduce biases such as the assumption that the statistical properties of the climate variables remain constant over time. This may not hold under changing climate conditions, where the relationships between variables can evolve. Moreover, while QM can adjust for biases in the overall distribution of climate variables, it may not effectively capture and correct biases in the frequency and intensity of extreme events. A possible solution to address this is to use machine-learning-based advanced bias correction techniques that account for non-stationarity between climate variables. Additionally, employing specialized statistical techniques for better handling of extreme events such as extreme value theory (EVT) can provide more reliable corrections for the tails of the distribution.

Soil data from the ISRIC-WISE dataset includes critical parameters like pH, bulk density, and organic carbon content. However, this dataset’s resolution and the interpolation assumptions used to estimate soil properties across grid cells may not capture local variations accurately, leading to potential inaccuracies. Possible ways to address these uncertainties could be using a hybrid approach where deterministic and stochastic modelling approaches are combined to capture both the predictable patterns and the random variations in soil properties. This can provide a more comprehensive understanding of soil variability. Additionally, ISRIC-WISE data can be fused with other datasets, such as remote sensing data, combined with more Field surveys and soil samplings, to improve spatial resolution and capture fine-scale variations.

Livestock population data from the Gridded Livestock of the World version 3 (GLW3) database, adjusted to align with FAO sub-national estimates, assumes a static distribution over time. This overlooks temporal changes in livestock distribution, which can skew population dynamics in the model. Land use and cover data, sourced from detailed layers provided by organizations like CILSS, USGS, and USAID, offer significant temporal and thematic detail. However, despite validation through various methods, these layers have an overall accuracy of around 73%, implying a margin of error that might affect land-use representation in the model. The SPAM harvest area dataset, used to estimate planted areas for crops such as maize, millet, and sorghum, also carries regional and crop-specific uncertainties. Assumptions about the static nature of crop distributions over time further complicate its accuracy. All these uncertainties in the data could be addressed to some extent by making use of high-resolution remote sensing data, combined with data-driven models and ground truthing. Thus, incorporating more frequent updates to temporal land use, livestock numbers, crop classification, and harvest area can better reflect changes over time and would improve the model estimations.

The diversity of varieties used in the study could have been improved and expanded^18,21^ and was largely limited by a lack of good quality datasets to allow calibration of the models for different cultivars, particularly LAI which is crucial for correct simulation of radiation capture and partitioning of water use between transpiration and evaporation. Another limitation is that the study considered only one crop model, and the models sometimes had different responses, as now demonstrated in several studies^3,4,18,48^. In summary, this research enhances our understanding of the model’s strengths and limitations in predicting agricultural outcomes and environmental dynamics. The findings underscore the intricate interplay between climate, crops, livestock, and emissions, highlighting the importance of accurate modeling for informed decision-making.

## Electronic supplementary material

Below is the link to the electronic supplementary material.

Supplementary Material 1

## Data Availability

The datasets used and/or analysed during the current study are available from the corresponding author upon reasonable request.

## References

[1] Alsafadi, K. et al. An evapotranspiration deficit-based drought index to detect variability of terrestrial carbon productivity in the Middle East. *Environ. Res. Lett.* **17** (1), 014051 (2022).

[2] Amole, T., Ayantunde, A., Balehegn, M. & Adesogan, A. T. Livestock feed resources in the west African sahel. *Agron. J.* **114**, 26–45. 10.1002/agj2.20955 (2022). 35910094 10.1002/agj2.20955 PMC9303701

[3] Asseng, S. et al. Uncertainty in simulating wheat yields under climate change. *Nat. Clim. Change*. **3**, 827–832 (2013).

[4] Bassu, S. et al. How do various maize crop models vary in their responses to climate change factors? *Global Change Biol.* **20**, 2301–2320 (2014). 10.1111/gcb.12520 24395589

[5] Batjes, N. H. Harmonized soil profile data for applications at global and continental scales: updates to the WISE database. *Soil Use Manag.* **25**, 124–127 (2009).

[6] Boone, R. B., Conant, R. T., Sircely, J., Thornton, P. K. & Herrero, M. Climate change impacts on selected global rangeland ecosystem services. *Glob Change Biol.* **24**, 1382–1393. 10.1111/gcb (2018). 10.1111/gcb.13995 29160927

[7] Cannon, A. J. Multivariate quantile mapping bias correction. An N-dimensional probability density function transform for climate model simulations of multiple variables. *Clim. Dyn.* **50**, 31–49. 10.1007/s00382-017-3580-6 (2018).

[8] Castellanos-Navarrete, A., Tittonell, P., Rufino, M. C. & Giller, K. E. Feeding, crop residue and manure management for integrated soil fertility management - a case study from Kenya. *Ag Syst.* **134**, 24–35 (2014).

[9] Sanou, C. L. et al. Larba Hubert Balima. Trends and impacts of climate change on crop production in Burkina Faso. *J. Water Clim. Change*. **14**, 2773–2787. 10.2166/wcc.2023.137 (2023).

[10] Cheng, M., McCarl, B. & Fei, C. Climate Change and Livestock Production: A literature review. *Atmosphere* **13**, 140 (2022).

[11] Cotillon, S. E. & Mathis, M. L. Mapping land cover through time with the Rapid Land Cover Mapper—Documentation and user manual: U.S. Geological Survey Open File Report 2017–1012, 23 p. (2017).

[12] Descheemaeker, K. et al. Climate change adaptation and mitigation in smallholder crop-livestock systems in sub-saharan Africa: a call for integrated impact assessments. *Reg. Environ. Change*. **16**, 2331–2343. 10.1007/s10113-016-0957-8 (2016).

[13] Descheemaeker, K., Zijlstra, M., Masikati, P., Crespo, O. & Homann-Kee Tui, S. Effects of climate change and adaptation on the 640 livestock component of mixed farming systems: a modelling study from semi-arid Zimbabwe. *Agric. Syst.* **159**, 282–295. 10.1016/j.agsy.2017.05.004 (2018).

[14] Dieng, D. et al. Multivariate bias-correction of high-resolution regional climate change simulations for West Africa: Performance and climate change implications. *Journal of Geophysical Research: Atmospheres*, 127; e2021JD034836. (2022). 10.1029/2021JD034836

[15] Dieng, D. et al. Evaluation of the COSMOCLM high-resolution climate simulations over West Africa. *J. Geophys. Research: Atmos.* **122**, 1437–1455. 10.1002/2016JD025457 (2017).

[16] Enders, A. et al. SIMPLACE – A versatile modelling and simulation framework for sustainable crops and agroecosystems. *in silico Plants*. diad006; 10.1093/insilicoplants/diad006 (2023).

[17] Erenstein, O. Crop Residue Mulching in Tropical and Semi-Tropical Countries: An Evaluation of Residue Availability and Other Technological Implications. *Soil & Tillage Research*. 67, 115–133; 10.1016/S0167-1987(02)00062-4 (2002).

[18] Faye, B. et al. Potential impact of climate change on peanut yield in Senegal, West Africa. *Field Crop Res.* **219**, 148–159. 10.1016/j.fcr.2018.01.034 (2018).

[19] Fitzpatrick, R. G. J. et al. What drives intensification of mesoscale convective systems over the west African sahel under climate change? *J. Clim.* **33**, 3151–3172 (2020).

[20] Gaiser, T. et al. Modeling biopore effects on root growth and biomass production on soils with pronounced sub soil clay accumulation. *Ecol. Model.* **256**, 6–15 (2013).

[21] Gbegbelegbe, S. et al. Nelson, G. Baseline simulation for global wheat production with CIMMYT mega-environment specific cultivars. *Field Crops Res.* **202**, 122–135 (2017).

[22] Giorgi, F. & Gutowski, W. J. Regional dynamical downscaling and the CORDEX initiative. *Annu. Rev. Environ. Resour.* **40**, 467–490 (2015).

[23] Glotter, M. et al. Evaluating the utility of dynamical downscaling in agricultural impacts projections. *Natl. Acad. Sci.* **111**, 8776–8781. 10.1073/pnas.1314787111 (2014). 10.1073/pnas.1314787111 PMC4066535 24872455

[24] Godber, O. F. & Wall, R. Livestock and food security: vulnerability to population growth and climate change. *Glob. Change Biol.*10.1111/gcb.12589 (2014). 10.1111/gcb.12589 PMC4282280 24692268

[25] Hausfather, Z., Peters, G. P. & Emissions The ‘business as usual’ story is misleading. *Nature* **577**, 618–620. 10.1038/d41586-020-00177-3 (2020a). 31996825 10.1038/d41586-020-00177-3

[26] Hausfather, Z. & Peters, G. P. RCP8.5 is a problematic scenario for near-term emissions. *Proc. Natl. Acad. Sci.* 117, 27791–27792; (2020). 10.1073/pnas. 20171 24117. 10.1073/pnas.2017124117 PMC7668049 33082220

[27] Heinzeller, D. et al. The WASCAL high-resolution regionalclimate simulation ensemble for West Africa: Concept, dissemination, and assessment. *Earth Syst. Sci. Data*. **10**, 815–835 (2018).

[28] Herrero, M., Thornton, P. K., Gerber, P. & Reid, R. S. Livestock, livelihoods and the environment: understanding the trade-offs. *Curr. Opin. Environ. Sustain.*10.1016/j.cosust.2009.10.003 (2009).

[29] Holzworth, D. P. et al. A. APSIM – Evolution towards a new generation of agricultural ´ systems simulation. *Environ. Model. Softw.* **62**10.1016/j.envsoft.2014.07.009 (2014).

[30] Jones, P. G. & Thornton, P. K. The potential impacts of climate change on maize production in Africa and Latin America in 2055. *Glob. Environ. Change*. **13**, 51–59 (2003).

[31] Kaptué, T. A. T., Roujean, J. L. & Faroux, S. ECOCLIMAP-II: an ecosystem classification and land surface parameter database of Western Africa at 1 km resolution for the Africa Monsoon Multidisciplinary Analysis (AMMA) project. *Remote Sens. Environ.* **114**, 961–976 (2010).

[32] Kersebaum, K. C. et al. Analysis and classification of data sets for calibration and validation of agro-ecosystem models. *Environ. Model. Softw.* **72**, 402–417. 10.1016/j.envsoft.2015.05.009 (2015).

[33] Koubodana, D. N. H., Diekkrüger, B., Näschen, K., Adounkpe, J. & Atchonouglo, K. Impact of the accuracy of land cover data sets on the accuracy of land cover change scenarios in the Mono River Basin, Togo, West Africa. *Int. J. Adv. Remote Sens. GIS*. **8** (1), 3073–3095 (2019).

[34] Lesnoff, M. *DynMod: a tool for demographic projections of tropical livestock populations under Microsoft Excel - User’s Manual - Version 1. ILRI Manuals and Guides*6 (CIRAD, 2008).

[35] Masikati, P. et al. Nhamo, N. Smart Technologies for Sustainable Smallholder Agriculture. ISBN 978-0-12-810521- (2017). 10.1016/B978-0-12-810521-4.00013-X

[36] Mueller, N. D. et al. Closing yield gaps through nutrient and water management. *Nature* **490**, 254–257. 10.1038/nature11420 (2012). 22932270 10.1038/nature11420

[37] Nikulin, G. et al. Precipitation climatology in an ensemble of CORDEX-Africa regional climate simulations. *J. Clim.* **25**, 6057–6078 (2012).

[38] Panitz, H. J., Dosio, A., Büchner, M., Lüthi, D. & Keuler, K. COSMO-CLM (CCLM) climate simulations over CORDEX-Africa domain: analysis of the ERA-Interim driven simulations at 0.44°and 0.22° resolution. *Clim. Dyn.* **42**, 3015–3038 (2014).

[39] Rahimi, J., Mutua, J. Y., Notenbaert, A. M., Dieng, D. & Butterbach-Bahl, K. Will dairy cattle production in West Africa be challenged by heat stress in the future? *Clim. Change*. **161**, 665–685 (2020).

[40] Rosenzweig, C. et al. The Agricultural Model Intercomparison and Improvement Project (AgMIP): protocols and pilot studies. *Agric. For. Meteorol.* **170**, 166–182. 10.1016/j.agrformet.2012.09.011 (2013).

[41] Rotter, R. P. & Van Keulen, H. Variations in yield response to fertilizer application in the tropics: II. Risks and opportunities for smallholders cultivating maize on Kenya’s arable land. *Agric. Syst.* **53**, 69–95. 10.1016/S0308-521X(96)00037-6 (1997).

[42] Rufino, M. C. et al. Network analysis of N flows 755 and food self-sufficiency—a comparative study of crop-livestock systems of the highlands of East and southern Africa. *Agric. For. Meteorol.* **200**, 233–284. 10.1016/j.agrformet.2014.09.016 (2015).

[43] Rusinamhodzi, L. et al. A meta-analysis of long-term effects of conservation agriculture on maize grain yield under rain-fed conditions. *Agron. Sustain. Dev.* **31**, 657–673. 10.1007/s13593-011-0040-2 (2011).

[44] Schlenker, W. & Lobell, D. Robust negative impacts of climate change on African agriculture. *Environ. Res. Lett.* **5**, 014010 (2010).

[45] Singh, P. et al. An assessment of yield gains under climate change due to genetic modification of pearl millet. *Sci. Total Environ.* **601**, 1226–1237 (2017). 28605840 10.1016/j.scitotenv.2017.06.002 PMC5536252

[46] Srivastava, A. K. et al. Cassava yield gap – A model-based assessment in Nigeria. *Front. Sustain. Food Syst.* **6**, 1058775. 10.3389/fsufs.2022.1058775 (2023).

[47] Srivastava, A. K., Mboh, C. M., Gaiser, T., Webber, H. & Ewert, F. Effect of sowing date distributions on simulation of maize yields at regional scale – a case study in Central Ghana, West Africa. *Agric. Syst.*10.1016/j.agsy.2016.05.012 (2016).

[48] Sultan, B., Ahmed, A. I., Faye, B. & Tramblay, Y. Lessnegative impacts of climate change on crop yields in West Africa in the new CMIP6 climate simulations ensemble. *PLOS Clim.* **2**, e0000263. 10.1371/journal.pclm.0000263 (2023).

[49] Sultan, B. et al. Assessing climate change impacts on sorghum and millet yields in the Sudanian and Sahelian savannas of West Africa. *Environ. Res. Lett.* **8**, 014040. 10.1088/1748-9326/8/1/014040 (2013).

[50] Tappan, G. G. et al. *West Africa Land Use Land Cover Time Series: U.S. Geological Survey Data Release* (U.S. Geological Survey, 2016).

[51] Tarawali, S., Herrero, M., Descheemaeker, K., Grings, E. & Blümmel, M. Pathways for sustainable development of mixed crop livestock systems: taking a livestock and pro-poor approach. *Livest. Sci.* **139**, 11–21. 10.1016/j.livsci.2011.03.003 (2011).

[52] Thornton, P. K. & Herrero, M. Adapting to climate change in the mixed crop and livestock farming systems in sub-saharan Africa. *Nat. Clim. Change*. **5**, 830–836. 10.1038/nclimate2754 (2015).

[53] Thornton, P. K., van de Steeg, J., Notenbaert, A. & Herrero, M. The impacts of climate change on livestock and livestock systems in developing countries: a review of what we know and what we need to know. *Agric. Syst.* **101**, 113–127. 10.1016/j.agsy.2009.05.002 (2009).

[54] Valbuena, D. et al. Identifying determinants, pressures and trade-offs of crop residue use in mixed smallholder farms in Sub-saharan Africa and South Asia. *Agric. Syst.* **134**, 107–118 (2015).

[55] van de Ven, G. W. J., de Ridder, N., van Keulen, H. & van Ittersum M. K. concepts in production ecology for analysis and design of animal and plant-animal production systems. *Agric. Syst.* **76**, 507–525. 10.1016/S0308-521X(02)00110-5 (2003).

[56] Van Gordon, M. M. Methods for Earth System Analysis in the West African Sahel: Land Cover and Climate Through Computational and Applied Sciences. PhD thesis. University of California, Berkeley (2018).

[57] Van Ittersum, M. K. et al. Yield gap analysis with local to global relevance—a review. *Field Crops Res.* **143**, 4–17. 10.1016/j.fcr.2012.09.009 (2013).

[58] van Vuuren, D. P. et al. The representative concentration pathways: an overview. *Clim. Change*. **109**, 5–31. 10.1007/s10584-011-0148-z (2011).

[59] Whitbread, A. M., Robertson, M. J., Carberry, P. S. & Dimes, J. P. How farming systems simulation can aid the development of more sustainable smallholder farming systems in southern Africa. *Eur. J. Agron.* **32**, 51–58 (2010).

[60] Yu, Q. et al. A cultivated planet in 2010–Part 2: the global gridded agricultural-production maps. *Earth Syst. Sci. Data*. **12** (4), 3545–3572 (2020).

