# Supplementary material for: Modelling mixed crop-livestock systems and climate impact assessment in sub-Saharan Africa
Source: Sci Rep. 2025 Jan 9;15:1399. doi: 10.1038/s41598-024-81986-8 (PMC11717939; doi:10.1038/s41598-024-81986-8)
Supplement: Supplementary file 1 — Supplementary Material 1 [file 41598_2024_81986_MOESM1_ESM.pdf]

Supplementary

Table S1: Comparison of simulated and observed average crop yields, aboveground biomass (AGB) in kg ha^{-1}, and Harvest Index (HI).

	Yield		MR	AGB		MR	HI		MR
	observed	*predicted*		*observed*	*predicted*		*observed*	*predicted*	
Millet	2130.0	2174.3	2.1	9400.0	9499.1	1.1	0.227	0.229	1.0
Maize	2519.2	2545.0	1.0	4845.3	5076.2	4.8	0.52	0.50	-3.6
Sorghum	1432.0	1540.0	7.5	6768.0	6903.2	2.0	0.21	0.22	5.4

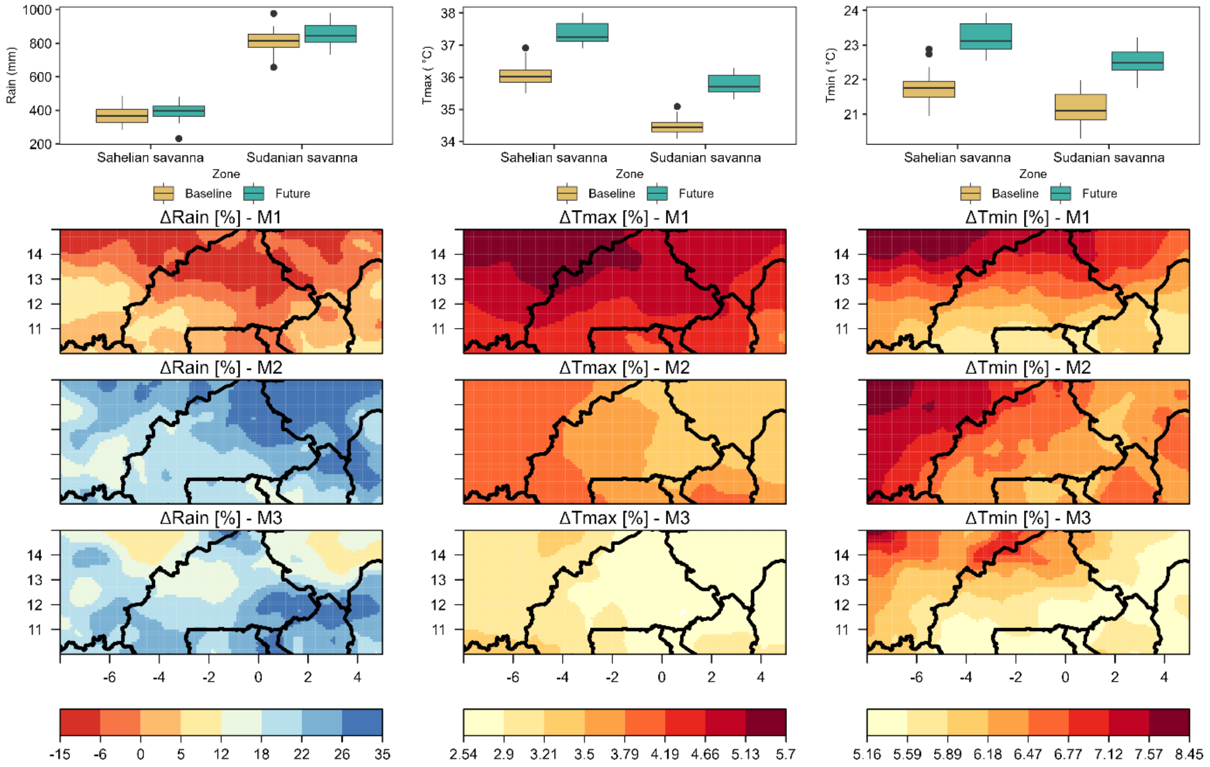

Figure S2: Boxplots of rainfall, maximum and minimum temperatures, for the historical (baseline) and future simulations in both Sahelian and Sudanian savanna regions for all GCMs (top panels); and the corresponding maps of the mean differences (%) between historical and future rainfall (ΔRain), maximum and minimum temperatures (ΔTmax and ΔTmin) for each GCM (M1, M2, M3). The solid black lines on the maps represent the territorial borders.

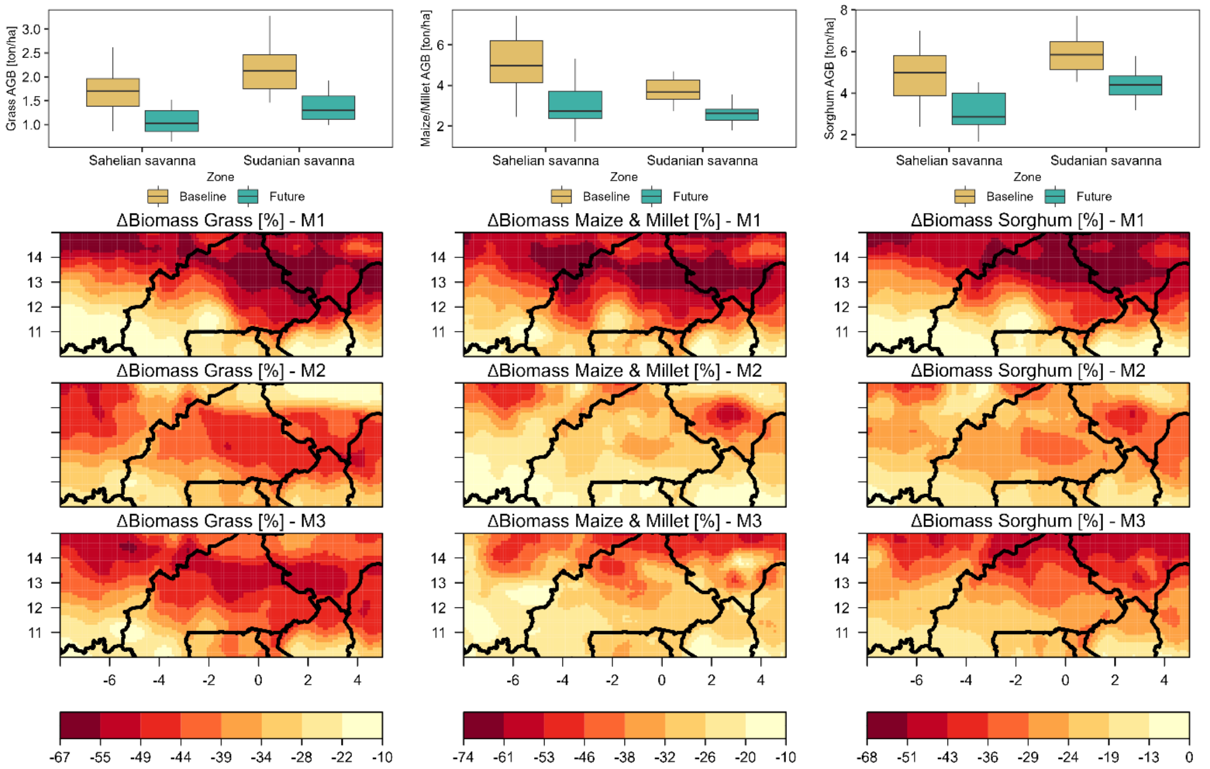

Figure S3: Boxplots of aboveground biomass (AGB) for grassland, maize and millet, and sorghum for the historical (baseline) and future simulations in both Sahelian and Sudanian savanna regions for all GCMs (top panels); and the corresponding maps of the mean differences (%) between historical and future aboveground biomass produced by the grassland, maize and millet, and sorghum for each GCM (M1, M2, M3). The solid black lines on the maps represent the territorial borders.

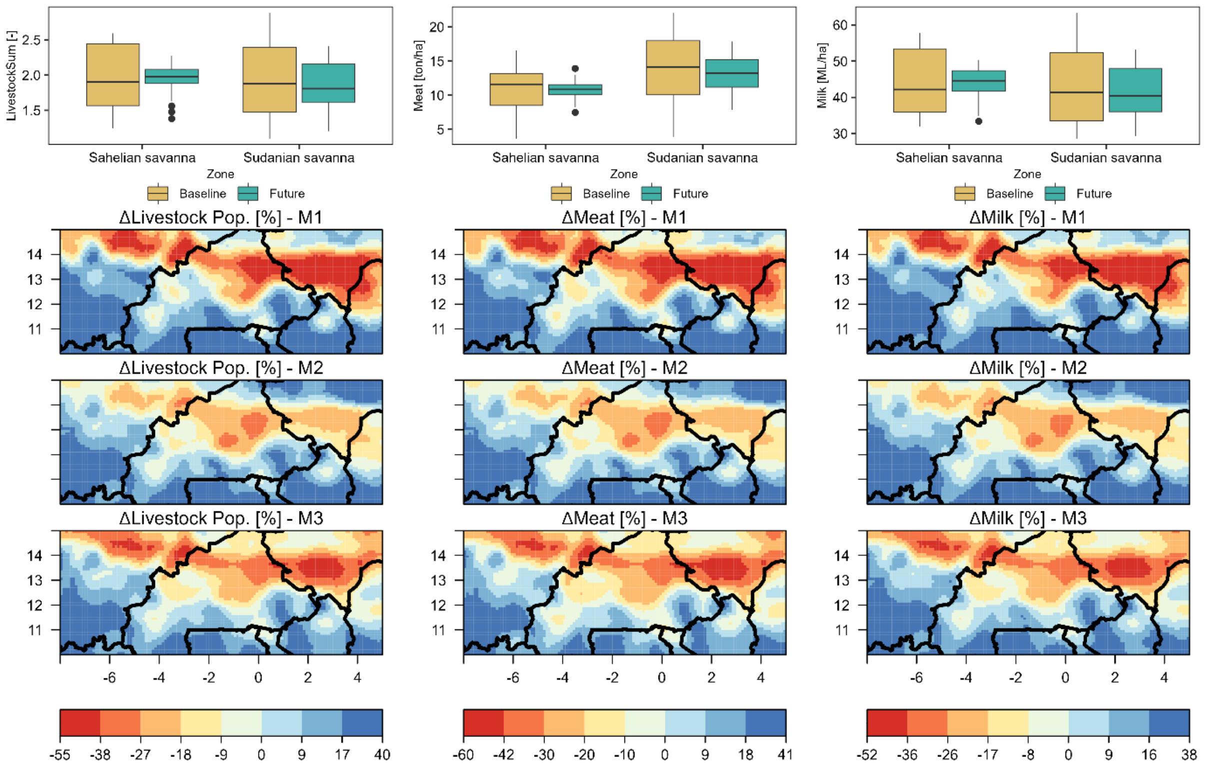

Figure S4: Boxplots of livestock population, meat, and milk for the historical (baseline) and future simulations in both Sahelian and Sudanian savanna regions for all GCMs (top panels); and the corresponding maps of the mean differences (%) between historical and future livestock population, meat, and milk for each GCM (M1, M2, M3). The solid black lines on the maps represent the territorial borders.

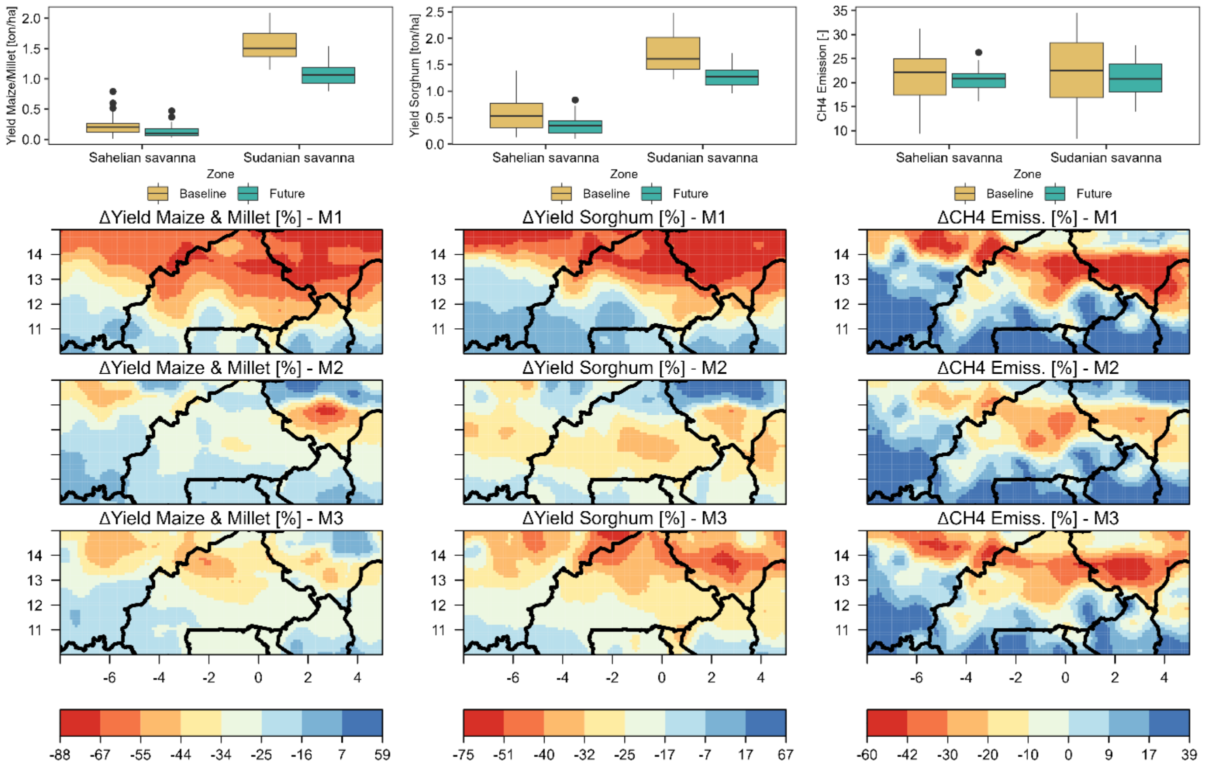

Figure S5: Boxplots of yield produced by the maize and millet, sorghum (in ton ha^{-1}), and methane emissions (ΔCH4) from the integrated livestock system for the historical (baseline) and future simulations in both Sahelian and Sudanian savanna regions for all GCMs (top panels); and the corresponding maps of the mean differences (%) between historical and future yield produced by the maize and millet, sorghum, and methane emissions (ΔCH4) from the integrated livestock system for each GCM (M1, M2, M3). The solid black lines on the maps represent the territorial borders.

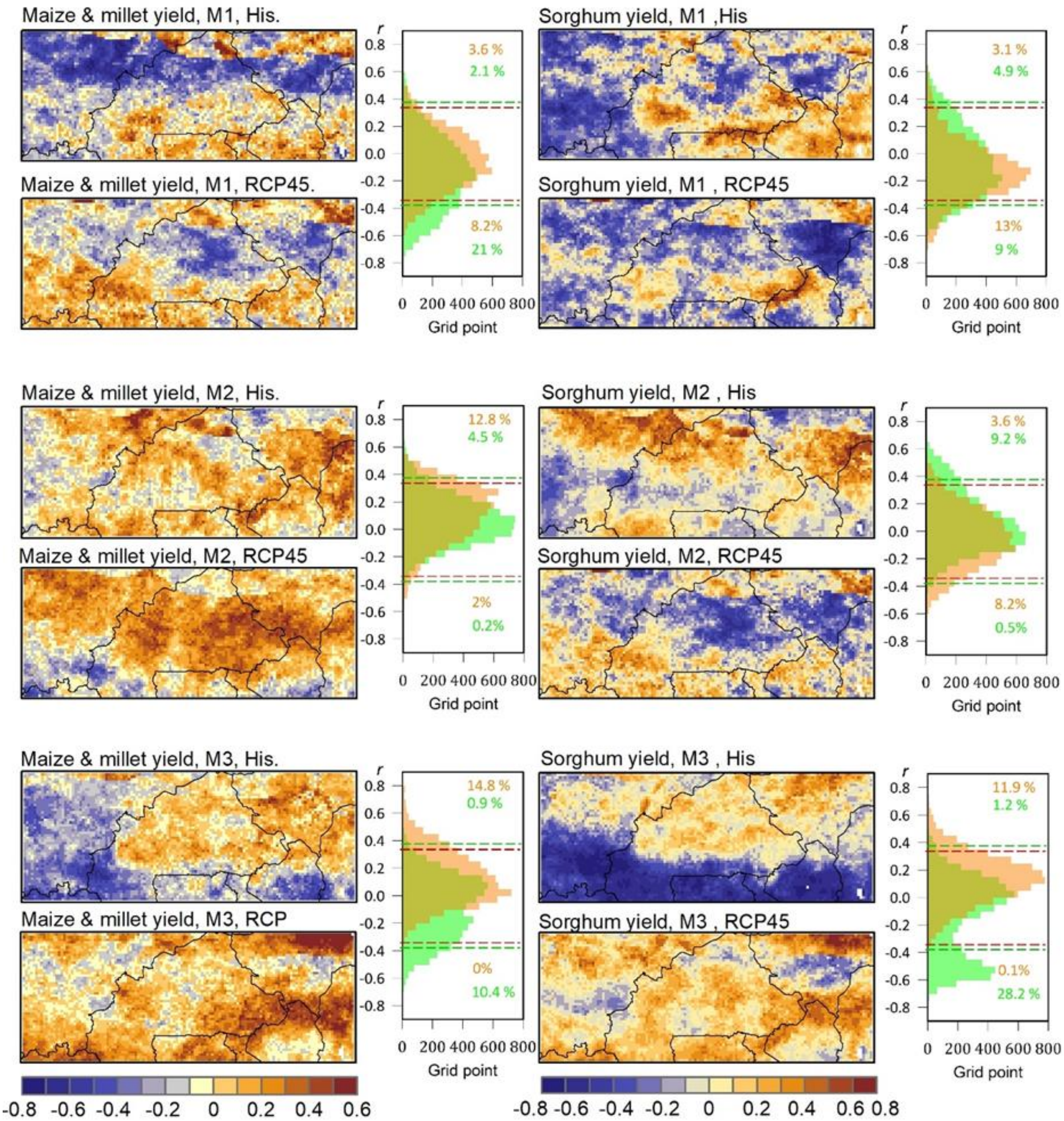

Figure S6: Spatial correlation between the crop yields and the annual sWSI for both current climate conditions ("His") and the projected climate change scenario (RCP4.5). In the histograms, the red and green dashed lines represent the critical values of correlation at a significant level of $p<0.05$ for the "His" and RCP4.5 datasets, respectively.

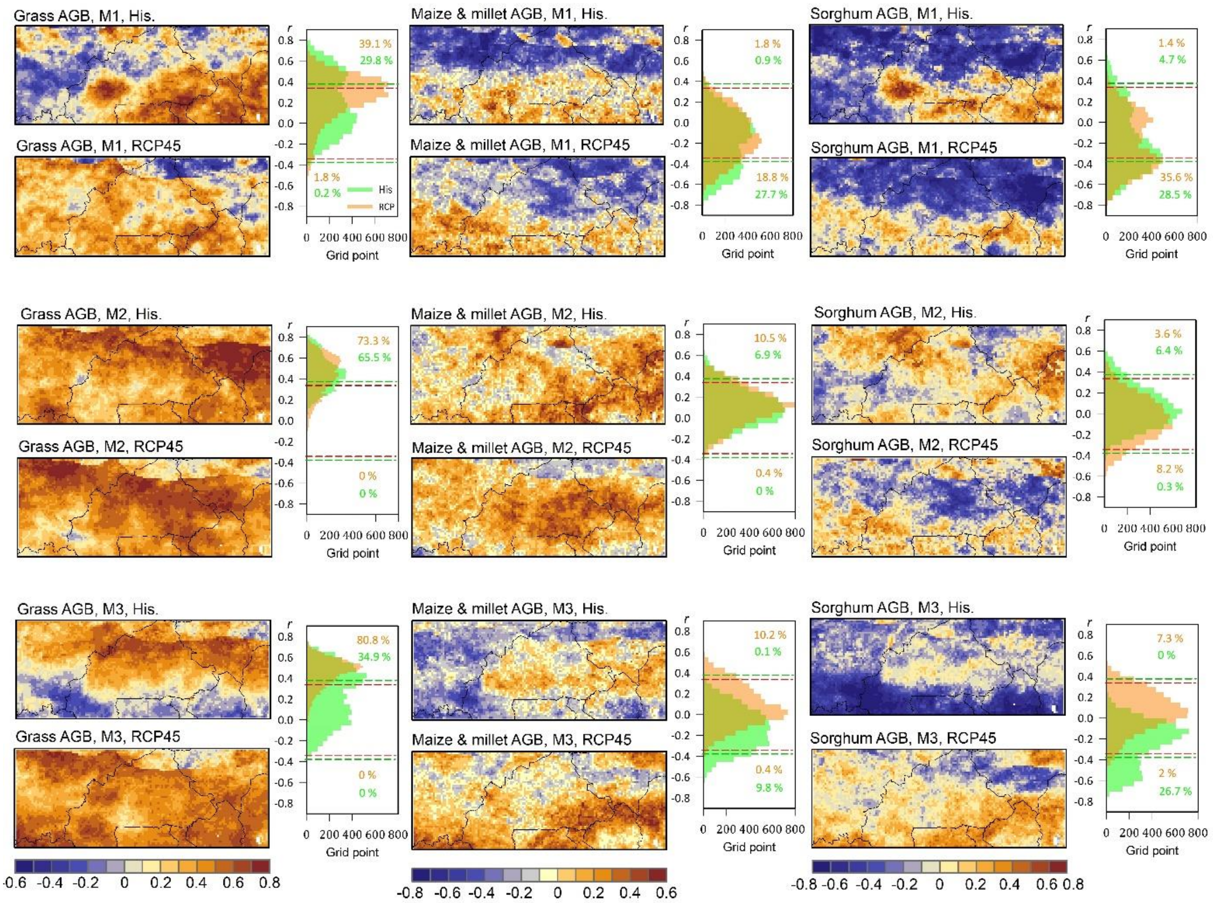

Figure S7: Spatial correlation between the AGB of grass and crops and the annual sWSI for both current climate conditions ("His") and the projected climate change scenario (RCP4.5). In the histograms, the red and green dashed lines represent the critical values of correlation at a significant level of $p<0.05$ for the "His" and RCP4.5 datasets, respectively.

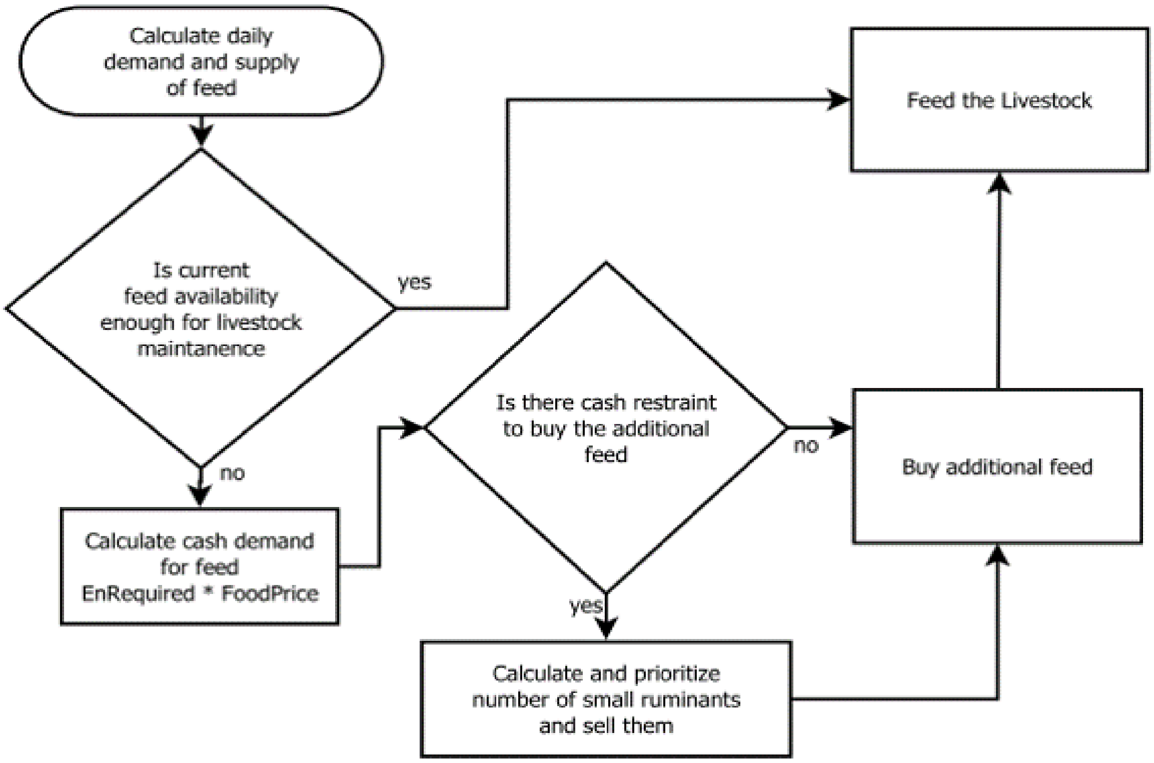

Figure S8: Schematic presentation of rule-based Livestock Sell out Management

Livestock Population Growth Simulation module:

The livestock population growth simulation module aims to calculate the daily change of animal numbers in six different age and gender classes (JuvenilesMale, SubAdultsMale, AdultsMale, JuvenilesFemale, SubAdultsFemale, AdultsFemale) (Table 1) based on birth rates ("Parturition" as Number of Birth per year per animal in the Age-gender class "AdultsFemale"), mortality rates in each of the Age-gender classes (e.g. "AdultFemaleMortailityFraction" per year), transition rate (see the "Gamma" factor down), Intake rate und offtake rate (in animals total). Keep in mind that there is no age or weight of the individual animal, just a count of the animals in each class and the transition process from one age class to another.

Table 1: Definition of animal classes according to gender and timespan (in years) staying the class and gender. The default values of the timespan can be modified by the user.

Males	Timespan in years (default values)	Females	Timespan (a) (default values)
JuvenilesMale	1	JuvenilesFemale	1
Sub-adultsMale	3	Sub-adultsFemale	3
AdultsMale	6	AdultsFemale	11

The calculation of the birth rate is based on the input parameter “Parturition”. The parturition can be modified by the “Prolification” fraction, which is the user-defined survival rate during birth. Then, the animals that survived the birth are partitioned into male and female by the user-defined “FemaleAtBirth” fraction. Finally, the birth rate is reduced in times of feed energy deficiency by the daily “ProductionNutritionIndex” which is calculated by the SimComponent “LivestockEnergyStress”.

The transition process from the age-gender classes juvenile to sub-adult and from sub-adult to adult is determined by the “gamma” factor, which is calculated by

Gamma = (pow(surv, d - 1) - pow(surv, d)) / (1 - pow(surv, d));

Where “surv” is the survival probability (as fraction 1 - mortality fraction – offtake fraction) and “d” is the user-defined timespan of an animal staying in a given age-gender class (in years).

The calculation of the daily change in animal numbers per Age-gender class is extended with feedback between the mortality factors and the deficiency of energy supply to the population triggered by the variable “MaintenanceNutritionIndex”, which is calculated by the SimComponent “LivestockEnergyStress” and the input parameter “cMaintenanceNutritionMortalityFactor.

mortalityFactor = (1 - maintenanceNutritionIndex) * MaintenanceNutritionMortalityFactor + maintenanceNutritionIndex;

Weight, Meat, and Milk Production simulation module:

This module is part of the DynMod Model (Lesnoff, 2008) to calculate the daily changes in live weight in each of the age-gender classes as defined in the “Livestock Population Growth simulation module” (JuvenilesMale, Sub-adultsMale, AdultsMale, JuvenilesFemale, Sub-adultsFemale, AdultsFemale). It contains the modules:

- Live weight (kg) per animal;
- Carcass yield (%), used for calculating the meat production;

The average weight of the animals in the respective age-gender classes is defined in Table 2.

Table 2: Average weight of animals in the six age-gender classes.

Males	g animal^{-1}	Females	g animal^{-1}
JuvenilesMale	54000	JuvenilesFemale	54000
Sub-adultsMale	138000	Sub-adultsFemale	133000
AdultsMale	244000	AdultsFemale	219000

There is no change in the age or weight of the individual animal. The changes in weight and meat are just based on the number of the animals in each age-gender class and the birth and transition processes from one age-gender class to another, which are calculated in the “Livestock Population Growth simulation module”

Thus, this module just multiplies the number of animals in each AgeGender class with the average live weight of the animals and then sums them up to obtain the total live weight of the population on a given day. The daily change of live weight in an age-gender class or the whole population can then be calculated from the weights on the current day and the weights on the previous day.

The daily available meat (“SumMeat”) is defined as the product of the live weight of the population (“SumWeight”) times the Carcass fraction, which is 0.47 by default. Selling of meat (“Takeoff”) cannot be done directly from the SumMeat in this module but always has to be done in the Livestock Population Growth simulation module, by selling animals (“Takeoff”) from age-gender classes and multiplying the number of sold animals with the average live weight in the respective age-gender class and with the carcass fraction. As the time step of the module is a day, the number of sold animals can be a decimal fraction.

The amount of daily available milk will be calculated as:

Total Milk produced = Adult Female Count * Parturition * Milk Offtake * Milking Days / 365

Total Milk produced: Milk produced in g day^{-1};

Adult Female Count: Decimal number of the adult female partition;

Parturition: Fraction of Birth per AdultFemale, default 0.5;

Milk Offtake: Amount of Milk per Animal and Day, default 1500 g;

Milking Days: Amount of days one animal gives milk, default 90 days.

This amount is reduced by the juvenile animal consumption as:

Total Milk consumed = (Juvenile Female + Juvenile Male) * Juvenile Consumption;

Total Milk consumed: Milk consumed by animals in g day^{-1};

Juvenile Female/Male: Decimal number of the juvenile male/female partition;

Juvenile_Consumption: Milk consumed by animals default 800 g day^{-1}.

The amount of milk exported is calculated as:

Total Milk exported = Total Milk produced – Total Milk consumed

For Methane emission, different algorithms are used:

(1) Methane emission = 20.7 * Dry Matter consumed

Methane emission: Enteric Fermentation Emission in g day^{-1}

Dry Matter consumed: from Energy consumption [Joule] / 18.1 [Joule g^{-1}] in g day^{-1}

(2) Methane emission = Total Animal Weight * Specific Methane emission

Total Animal Weight: Sum of weight of animals of the age gender classes in g

Specific Methane emission: 0.000018 [g J^{-1}] * an age gender class specific conversion fraction of energy in feed between 0.045 and 0.065
